# Epigenetic imprinting alterations as effective diagnostic biomarkers for early-stage lung cancer and small pulmonary nodules

**DOI:** 10.1186/s13148-021-01203-5

**Published:** 2021-12-14

**Authors:** Jian Zhou, Tong Cheng, Xing Li, Jie Hu, Encheng Li, Ming Ding, Rulong Shen, John P. Pineda, Chun Li, Shaohua Lu, Hongyu Yu, Jiayuan Sun, Wenbin Huang, Xiaonan Wang, Han Si, Panying Shi, Jing Liu, Meijia Chang, Maosen Dou, Meng Shi, Xiaofeng Chen, Rex C. Yung, Qi Wang, Ning Zhou, Chunxue Bai

**Affiliations:** 1 Department of Pulmonary Medicine, Shanghai Respiratory Research Institute, Zhongshan Hospital, Fudan University, Shanghai, 200032 China; 2 Shanghai Engineering Research Center of Internet of Things for Respiratory Medicine, Shanghai, 200032 China; 3 Epigenetics Lab, Chinese Alliance Against Lung Cancer, 6th Floor, Building 5, No.66, Jinghuidongdao Road, Wuxi, 214135 Jiangsu China; 4 Department of Respiratory Medicine, The Second Hospital Affiliated to Dalian Medical University, Dalian, 116044 Liaoning China; 5 Department of Respiratory Medicine, The Affiliated Zhongda Hospital of Southeast University, Nanjing, 210009 Jiangsu China; 6 Department of Pathology, Ohio State University Wexner Medical Center, Columbus, OH 43210 USA; 7 Department of Pathology, Changzheng Hospital, Navy Medical University, Shanghai, 200003 China; 8 Department of Respiratory Endoscopy and Respiratory and Critical Care Medicine, Shanghai Chest Hospital, Shanghai Jiao Tong University, Shanghai, 200030 China; 9 Department of Pathology, Nanjing First Hospital, Nanjing, 210006 Jiangsu China; 10 Department of Pathology, The Affiliated Yantai Yuhuangding Hospital, Qingdao University, Yantai, 264000 Shandong China; 11 Department of Cardiothoracic Surgery, Huashan Hospital, Fudan University, Shanghai, 200040 China; 12 Department of Oncology, Johns Hopkins University School of Medicine, Baltimore, MD 21207 USA

**Keywords:** Epigenetics, Genomic imprinting, Cancer biomarkers, In situ hybridization, Lung cancer early diagnosis, Pulmonary nodules

## Abstract

**Background:**

Early lung cancer detection remains a clinical challenge for standard diagnostic biopsies due to insufficient tumor morphological evidence. As epigenetic alterations precede morphological changes, expression alterations of certain imprinted genes could serve as actionable diagnostic biomarkers for malignant lung lesions.

**Results:**

Using the previously established quantitative chromogenic imprinted gene *in situ* hybridization (QCIGISH) method, elevated aberrant allelic expression of imprinted genes *GNAS*, *GRB10*, *SNRPN* and *HM13* was observed in lung cancers over benign lesions and normal controls, which were pathologically confirmed among histologically stained normal, paracancerous and malignant tissue sections. Based on the differential imprinting signatures, a diagnostic grading model was built on 246 formalin-fixed and paraffin-embedded (FFPE) surgically resected lung tissue specimens, tested against 30 lung cytology and small biopsy specimens, and blindly validated in an independent cohort of 155 patients. The QCIGISH diagnostic model demonstrated 99.1% sensitivity (95% CI 97.5–100.0%) and 92.1% specificity (95% CI 83.5–100.0%) in the blinded validation set. Of particular importance, QCIGISH achieved 97.1% sensitivity (95% CI 91.6–100.0%) for carcinoma in situ to stage IB cancers with 100% sensitivity and 91.7% specificity (95% CI 76.0–100.0%) noted for pulmonary nodules with diameters ≤ 2 cm.

**Conclusions:**

Our findings demonstrated the diagnostic value of epigenetic imprinting alterations as highly accurate translational biomarkers for a more definitive diagnosis of suspicious lung lesions.

**Supplementary Information:**

The online version contains supplementary material available at 10.1186/s13148-021-01203-5.

## Background

Lung cancer is the leading contributor of cancer deaths [1]. Compared to late-stage lung cancer, early-stage lung cancer showed better prognosis and longer survival [2]. Low-dose computed tomography (LDCT) screening has made great contribution to the early discovery of lung cancer and reducing lung cancer mortality [3]. However, the presurgical diagnosis of early-stage lung cancer from standard diagnostic biopsies is still challenging because of insufficient tumor morphological evidence to make a definitive pathological diagnosis [4]. Several genetic [5–7] and epigenetic biomarkers [8–10] have been developed for early cancer detection. However, the reliability and efficiency of these biomarkers have yet to be optimized for clinical applications [11].

As an important epigenetic regulation in mammalian embryo development, genomic imprinting plays important roles in cancers [12, 13]. In normal post-natal somatic cells, imprinted genes are “silenced”, that is mono-allelically expressed either from the maternal or paternal allele, while in cancers, some silenced imprinting genes’ copies could be reactivated, leading to expressions from both alleles. The loss of monoallelic gene regulation is named loss of imprinting (LOI), and has been previously found in various human cancers [13–18]. In addition to LOI, amplifications of the activated copies of imprinted genes without affecting the methylation of the silenced copy have also been observed in multiple cancer cell lines [19]. In both cases, the imprinted genes could be expressed in two or more transcription sites instead of one. Therefore, the increased number of transcription site detections of imprinted genes in the cell nuclei could be used as potential cancer biomarkers. The nascent RNA or pre-mRNA in situ hybridization (ISH) method targeting the short-lived introns can be used to visualize and label these transcription sites [20–23], and have been widely applied to study the transcriptional regulations of both imprinted genes [24–27] and non-imprinted genes [28, 29]. In our previous study, we have adopted this intron-targeted labeling approach and developed an objective quantification of epigenetic imprinting alterations through the biallelic (BAE), multiallelic (MAE) and total (TE) expression measures which we termed as quantitative chromogenic imprinted gene *in situ* hybridization (QCIGISH) [30]. Based on the elevated BAE, MAE and TE signatures observed for various cancers over benign lesions, we formulated a statistical malignancy prediction model and identified *GNAS*, *GRB10* and *SNRPN* as effective diagnostic biomarkers in ten different cancer types, including lung cancer [30]. Despite the preliminary model achieving 92% sensitivity and 88% specificity for lung cancer diagnosis, opportunities to further advance the diagnostic performance of QCIGISH in clinical applications need to be explored.

In this study, aiming to develop a lung cancer-specific diagnostic model with improved accuracy, we expanded the imprinted gene panel with a fourth imprinted gene minor histocompatibility antigen H13 (*HM13*). As amplification of *HM13* locus has been previously reported in several lung cancer cell lines [19], it is very likely to demonstrate multiallelic expressions using our QCIGISH method. We conducted a differential analysis and statistical evaluation of the QCIGISH epigenetic imprinting alteration measurements obtained for the normal, benign and malignant lung tissue specimens. To pathologically confirm the relationship between epigenetic imprinting and carcinogenesis, we performed a comparative examination between the imprinting signatures obtained from QCIGISH and morphological characteristics determined through histologic staining. From the alteration patterns, we developed a diagnostic grading model for lung tissue specimens, tested and validated the model using cytology and small biopsy specimens obtained via bronchoscopy or transthoracic CNB, and evaluated the results in comparison with standard diagnostic biopsies. We particularly investigated the diagnostic value of epigenetic imprinting biomarkers in effectively providing clearer malignancy differentiation especially for early-stage lung cancers, with the objective of improving the accuracy of standard diagnostic biopsies for lung lesions.

## Results

### Patient characteristics

**Fig. 1:**
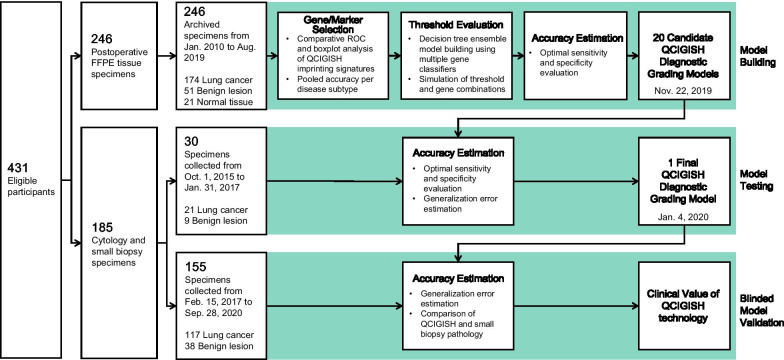
Workflow showing the study design and analysis steps of model building, testing and validation

Clinicopathological characteristics between different patient groups in the imprinted gene screening (30 lung cancers and 30 benign lesions); model building and marker pre-selection (174 lung cancers, 51 benign lesions and 21 normal controls); model testing (21 lung cancers and 9 benign lesions) and model validation (117 lung cancers and 38 benign lesions) cohorts are described in Fig. 1 and Additional file 1: Figs. S1-2 are comparatively analyzed, statistically evaluated and summarized in Table 1 and Additional file 2: Table S1.

**Table 1:** Baseline characteristics of study population

	Model building set (*n* = 246)				Model testing set (*n* = 30)			Model validation set (*n* = 155)		
	Normal^a^	Benign	Malignant	*p*	Benign	Malignant	*p*	Benign	Malignant	*p*
	(*n* = 21)	(*n* = 51)	(*n* = 174)		(*n* = 9)	(*n* = 21)		(*n* = 38)	(*n* = 117)	
Age				< 0.001			0.700			0.001
Median	52	57	62		62	62		61	64	
IQR	45–57	49–63	53–67		49–77	56–66		52–63	58–71	
Sex (%)				0.467			0.640			0.692
Male	14 (66.7%)	28 (54.9%)	111 (63.8%)		6 (66.7%)	17 (81.0%)		25 (65.8%)	81 (69.2%)	
Female	7 (33.3%)	23 (45.1%)	63 (36.2%)		3 (33.3%)	4 (19.0%)		13 (34.2%)	36 (30.8%)	
Sample type										
Surgically resected tissue specimen	21	51	174							
Small biopsy specimen					9	21		38	36	
Cytology specimen									81	
Histologic characteristics no										
Normal	21									
PC		10								
PSP		13								
TB		10			2			7		
COP		10						2		
PIP		3								
Non-TB infections					3			8		
Inflammation		3			4			19		
Granuloma		2						1		
Hamartoma								1		
AdC			94			9			61	
SqCC			76			6			28	
AdSqLC			2						2	
LCC			1							
SCLC						5			23	
Carcinoma of unknown primary			1			1			3	
Nodule size				^e^			^e^			^e^
< 0.8 cm			7		2			10	1	
≥ 0.8–2.0 cm		3	35		1	1		2	20	
> 2.0–3.0 cm			48			9		3	32	
> 3.0–5.0 cm		1	52			4		4	42	
> 5.0 cm			28			6			18	
Unclear LDCT features^b^	21	44			5			12	1	
Not specified^c^		3	4		1	1		7	3	
Smoking status^d^ (%)				0.001			^e^			0.006
Current smoker	3 (14.3%)	3 (5.9%)	56 (32.2%)		1 (11.1%)	6 (28.6%)		7 (18.4%)	48 (41.0%)	
Former smoker	1 (4.8%)	1 (2.0%)	11 (6.3%)		0 (0.0%)	3 (14.3%)		2 (5.3%)	11 (9.4%)	
Non-smoker	15 (71.4%)	40 (78.4%)	90 (51.7%)		1 (11.1%)	8 (38.1%)		16 (42.1%)	40 (34.2%)	
Not specified	2 (9.5%)	7 (13.7%)	17 (9.8%)		7 (77.8%)	4 (19.0%)		13 (34.2%)	18 (15.4%)	

PC, Pulmonary cryptococcosis. PSP, pulmonary sclerosing pneumocytoma. TB, pulmonary tuberculosis. COP, cryptogenic organizing pneumonia. PIP, pulmonary inflammatory pseudotumor. AdC, adenocarcinoma. SqCC, squamous cell carcinoma. AdSqLC, adenosquamous lung carcinoma. LCC, large cell carcinoma. SCLC, small cell lung cancer

^a^Normal tissue specimens were resected adjacent to the benign lesions

^b^No typical nodule under LDCT

^c^Nodule sizes not recorded by doctors

^d^Cases classified as current or former smokers were combined into a single category prior to analysis to comply with the statistical test requirements

^e^No analysis proceeded since data transformations applied failed to meet the statistical test requirements

### Evaluation of candidate imprinted gene biomarkers

**Fig. 2:**
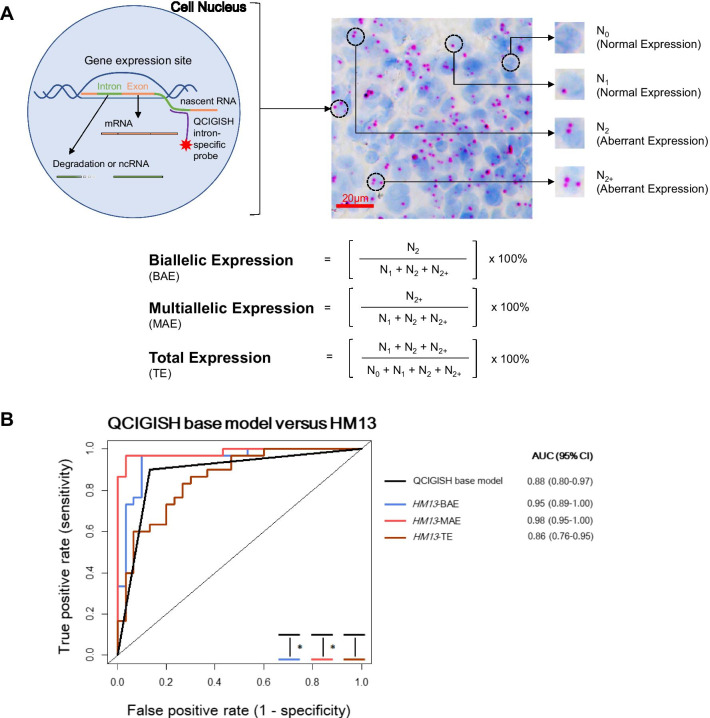
Principle of QCIGISH technology and novel imprinted gene evaluation. **A** Illustration showing the QCIGISH principle and the respective equations used for calculating BAE, MAE and TE measurements. The QCIGISH method targets the non-coding intronic nascent RNAs to visualize the transcription loci of imprinted genes in the cell nuclei. Blue components in the image are cell nuclei stained using hematoxylin. The distinct red or brown dots represent the detected gene-expressing sites. The different allelic expressions of imprinted genes are quantified based on the transcription signals. Aberrant expressions for abnormal cells exhibit two or more dots, while normal cells contain no to a single dot. **B** ROC curves showing the significant differences in the AUC values determined for the BAE and MAE of *HM13* as compared to the QCIGISH binary classification model during the imprinted gene selection study. *, significant differences between AUC values, *p* < 0.05

To evaluate the diagnostic performance of the candidate imprinted gene *HM13* against the *GNAS*, *GRB10* and *SNRPN* panel, we performed a random sampling of 30 tissue specimens each stratified for both benign and malignant subgroups from the model building set (Fig. 1 and Additional file 2: Table S1). QCIGISH was applied on the 60 samples to determine the BAE, MAE and TE measurements for all four genes (Fig. 2A). Using the expression status of the imprinted genes *GNAS*, *GRB10* and *SNRPN*, malignancy predictions for the samples were obtained using the QCIGISH binary classification model developed in our previous study [30]. The receiver operating characteristic (ROC) areas under the curve (AUC) of the BAE, MAE and TE measurements for the imprinted gene *HM13* were individually compared to the ROC AUC of the binary classification model. Significantly higher AUC values were only observed for both MAE (*p* = 0.008) and BAE (*p* = 0.044) except TE (*p* = 0.511) after evaluating the diagnostic performance of *HM13* against the previous binary classification model which combined the *GNAS*, *GRB10* and *SNRPN* genes (Fig. 2B and Additional file 2: Table S2). These findings substantiated the expansion of the *GNAS*, *GRB10* and *SNRPN* multi-marker panel to four imprinted genes including *HM13*.

### Differential epigenetic imprinting alteration signatures in lung tissue specimens

For the subsequent evaluation to further investigate the most efficient imprinting markers among the four-gene panel, elevated allelic expression patterns for the 174 lung cancers were observed from the heatmap analysis as compared to the 51 benign lesions and 21 normal controls (Additional file 1: Fig. S3A). Statistical evaluation of the BAE, MAE and TE status between these groups demonstrated a substantial increase in imprinting alterations (all *p* < 0.05) for the malignant cases as compared to both benign and normal samples (Additional file 1: Fig. S3B, Additional file 2: Table S3–S6). Significantly higher BAE and TE (all *p* < 0.05) were also observed for benign lesions as compared to normal controls (Additional file 1: Fig. S3B, Additional file 2: Table S3–S6).

**Fig. 3:**
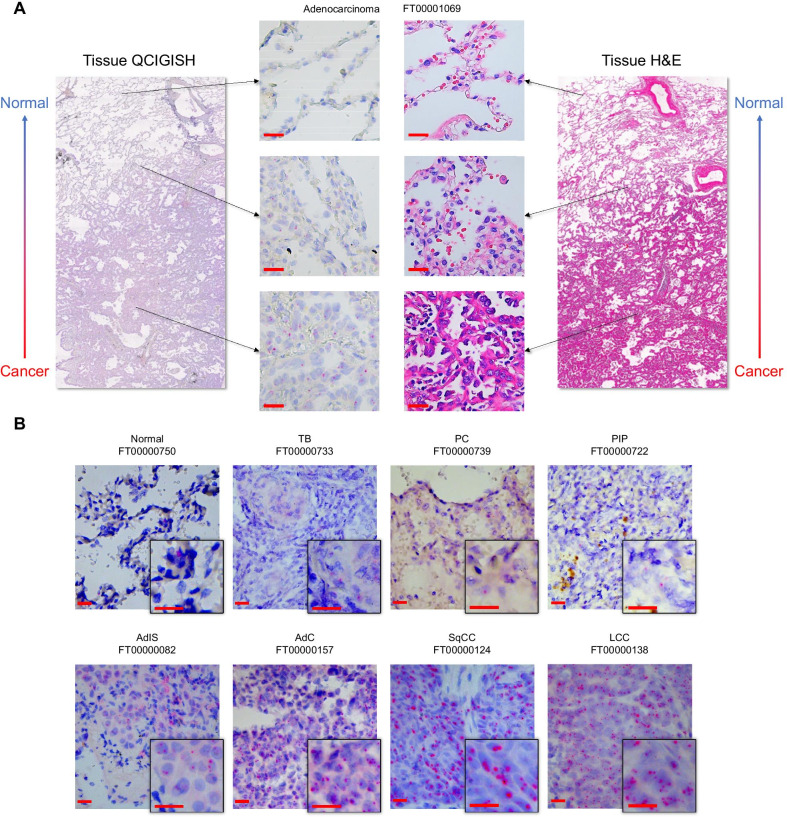
Photomicrographs showing the pathological assessment and confirmation of QCIGISH results. **A** Comparative analysis of QCIGISH and H&E staining applied on serially resected specimens from the same tissue block of an adenocarcinoma showing increasing imprinted gene *SNRPN* expression alterations in normal, paracancerous and cancer regions, respectively. The typical normal, paracancerous and cancer regions were all magnified for both QCIGISH and H&E staining. **B** Illustrated examples showing the visualized allelic expression of imprinted gene *GNAS* in resected tissue sections of benign lesion and lung cancer subtypes in the model building set. TB, pulmonary tuberculosis. PC, pulmonary cryptococcosis. PIP, pulmonary inflammatory pseudotumor. AdIS, adenocarcinoma in situ. AdC, adenocarcinoma. SqCC, squamous cell carcinoma. LCC, large cell carcinoma. Scale bar, 20 μm

Elevated imprinting alterations were pathologically confirmed to be associated with tissue morphology. As illustrated in Fig. 3A, increasing aberrant imprinting signatures were observed between the normal, paracancerous and malignant regions on the same tissue section. Further analysis in different tissue sections showed clear differences in the allelic expression status of imprinted genes between benign and malignant cases (Fig. 3B). Imprinting alterations were visually detected to a greater extent for lung cancers, with elevated expressions observed as early as adenocarcinoma in situ, effectively distinguishing lung malignancy from benign lesions from a pathological perspective.

### QCIGISH lung cancer diagnostic grading model building and testing

From the comparative analysis of the malignancy discrimination between imprinting alteration markers, MAE consistently demonstrated higher ROC AUC (0.87 to 0.94) and was the best marker across all genes as compared to BAE (0.84–0.93) and TE (0.78–0.86) (Additional file 1: Fig. S4). In addition, when applying optimal thresholds to dichotomize BAE, MAE and TE into positive and negative categories (Additional file 2: Table S7), MAE demonstrated good specificity and sensitivity for all benign lung lesion subtypes and lung cancer subtypes included in this study (Additional file 1: Fig. S5). Therefore, we identified MAE as the most effective imprinting biomarker over BAE and TE. As each gene demonstrated distinct diagnostic efficacies across the different benign lesion and cancer subtypes, MAE from all four genes were used during diagnostic model building.

We subsequently developed the classification model for distinguishing lung malignancy on the basis of the MAE imprinting alteration signatures from the prior analysis. We adopted the decision tree ensemble model structure from our previous study which combined individual gene classifiers to create more robust diagnostic predictions [30] but upgraded the malignancy classification system from two to five levels and only used the MAE status for each gene (Additional file 1: Fig. S6 and Fig. S7). Through a simulation study of different threshold combinations, twenty candidate models with equally optimal sensitivity and specificity using top one grade or top two grades were determined (Additional file 1: Fig. S8 A–D, Additional file 2: Table S8 and S9).

The twenty candidate models were further tested in an independent set of cytology and small biopsy samples obtained via bronchoscopy or transthoracic CNB to determine the optimal threshold for final model. With thresholds 1 to 4 set at 81% specificity, 98% specificity, 46% sensitivity and 40% sensitivity targets, respectively, the model using the top two highest grades demonstrated the best diagnostic performance and was determined as the final model, achieving 95.2% sensitivity (95% CI 86.1–100.0%) and 100.0% specificity in the test set, over the model using top one grade [90.5% sensitivity (95% CI 77.9–100.0%), 100.0% specificity] (Additional file 1: Fig. S8E and Additional file 2: Table S10). Moreover, the model using two highest grades effectively classified a considerable subset of benign samples into QCIGISH-negative despite having one gene categorized as grade II with minimal number of malignant samples misclassified as QCIGISH-negative (Additional file 1: Fig. S9). The final QCIGISH diagnostic grading model was locked on January 4, 2020, with the process flow and threshold values for the individual genes summarized in Additional file 1: Figure S10 and Additional file 2: Table S11, respectively.

### QCIGISH lung cancer diagnostic grading model validation in lung cytology and small biopsy specimens

**Fig. 4:**
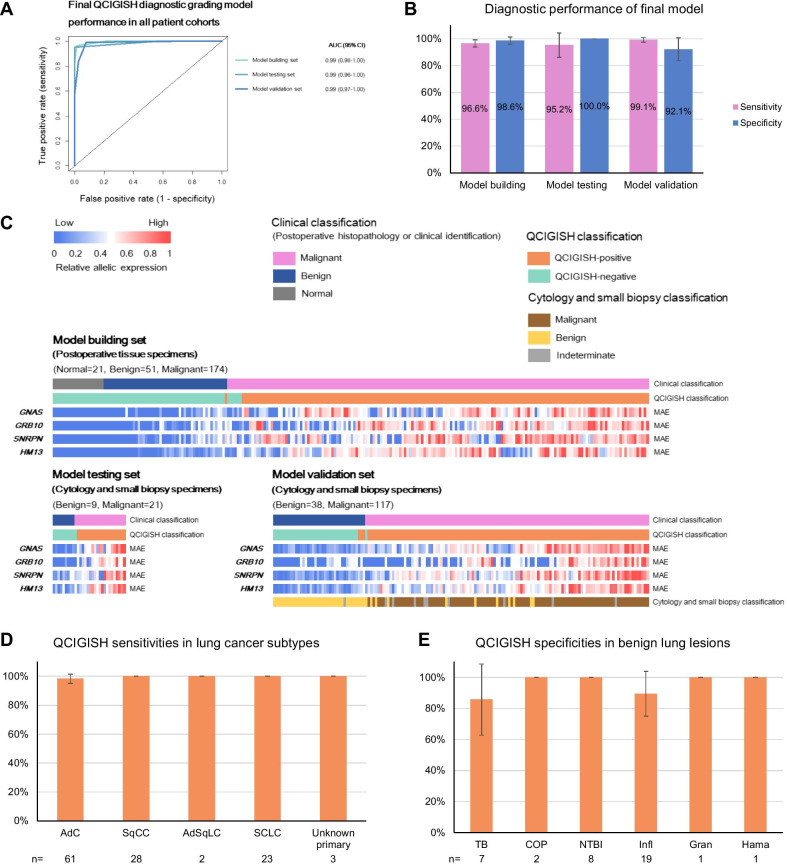
QCIGISH diagnostic performance in benign lesions and lung cancers. **A** ROC chart showing the AUC performance of the QCIGISH diagnostic grading model in the model building, testing and validation sets. **B** Computed sensitivities and specificities of the final QCIGISH diagnostic grading model in the model building, testing and validation sets. **C** Heatmap analysis plot showing the elevated multiallelic expression patterns for the lung cancers as compared to the benign lesions for the cytology and small biopsy specimens from both model testing and validation sets. **D** Analysis showing high sensitivities of QCIGISH for the diagnosis of different lung cancer subtypes. **E** Analysis showing high specificities of QCIGISH for the diagnosis of different benign lung lesions. AdC, adenocarcinoma. SqCC, squamous cell carcinoma. AdSqLC, adenosquamous lung carcinoma. SCLC, small cell lung cancer. TB, pulmonary tuberculosis. COP, cryptogenic organizing pneumonia. NTBI, non-TB infections. Infl, inflammation. Gran, granuloma. Hama, hamartoma. Error bars on the bar charts showed the 95% CI

We blindly validated the final QCIGISH diagnostic grading model in an independent cohort of 155 patients achieving an overall sensitivity of 99.1% (116/117, 95% CI 97.5–100.0%) and specificity of 92.1% (35/38, 95% CI 83.5–100.0%) with ROC AUC of 0.99 (95% CI 0.97–1.00) (Fig. 4A, B). The QCIGISH classification results were consistent with the clinical diagnoses which replicated those of the model building and test sets (Fig. 4A–C and Additional file 1: Fig. S11). Consistently high sensitivities were noted for the different NSCLC subtypes including adenocarcinoma (AdC) (98.4% = 60/61, 95% CI 95.2–100.0%), squamous cell carcinoma (SqCC) (100.0% = 28/28) and adenosquamous carcinoma (AdSqC) (100.0% = 2/2) (Fig. 4D and Additional file 2: Table S12). Despite having developed the diagnostic grading model particularly from NSCLC cases, QCIGISH was also highly effective for differentiating small cell lung cancer (SCLC) samples with 100.0% sensitivity (23/23) (Fig. 4D and Additional file 2: Table S12). The specificity of QCIGISH was also consistently high across benign lung lesions (85.7% for TB, 89.5% for inflammation and 100.0% for COP, non-TB infections, granuloma and hamartoma, Fig. 4E and Additional file 2: Table S12). Further analysis also showed equally high sensitivities (87.5–100.0%) and specificities (81.3–100%) with respect to patients’ clinical characteristics including gender and age, except for a slightly lower specificity for non-smokers than current or former smokers (Additional file 2: Table S13).

### Diagnostic performance comparison between the QCIGISH method and cytology and small biopsy pathology

**Fig. 5:**
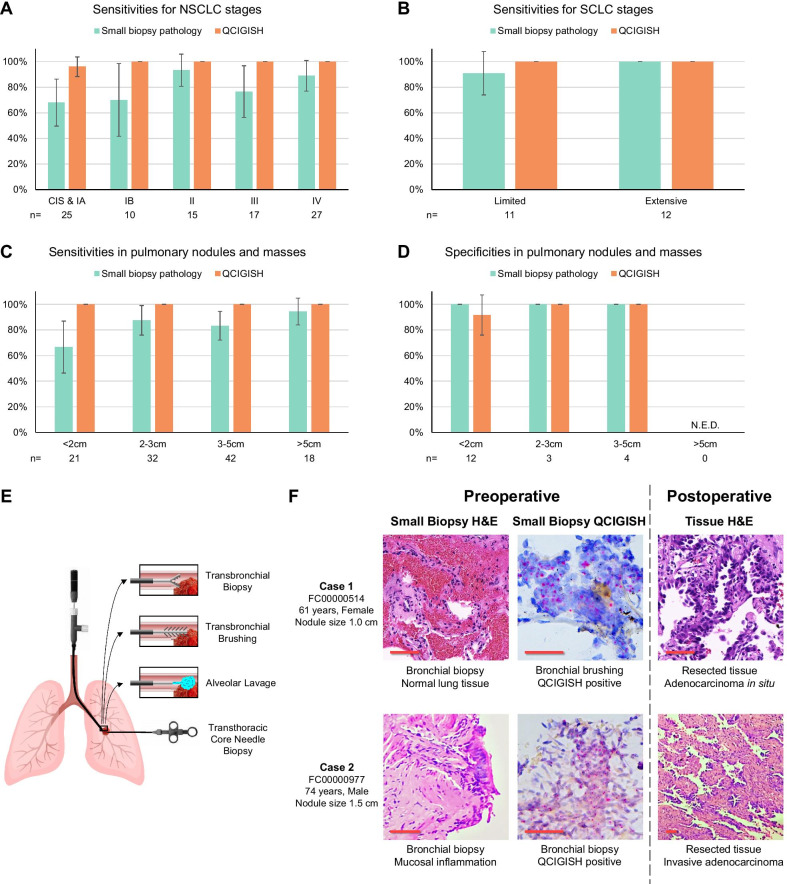
Comparison of the accuracies of QCIGISH and current small biopsy pathology for lung cancer diagnosis. **A** Analysis showing improved or comparable sensitivities of QCIGISH over small biopsy pathology for different stages of NSCLC. **B** Analysis showing improved or comparable sensitivity of QCIGISH over small biopsy pathology for different stages of SCLC. **C** Analysis showing improved or comparable sensitivities of QCIGISH over small biopsy pathology for pulmonary nodules and masses. **D** Analysis showing comparable specificities of QCIGISH with small biopsy pathology for pulmonary nodules and masses. **E** List of clinically available minimally invasive sampling procedures for lung lesions. **F** An illustrated example of clinical cases both positively classified as lung cancer by QCIGISH but were diagnosed as benign by small biopsy pathology. Surgical histopathology or clinical diagnosis with 2-year follow-up were used as golden standard. CIS, carcinoma in situ. N.E.D., no enough data. Error bars on the bar charts showed the 95% CI. Scale bar, 50 μm

Comparing with standard cytology and small biopsy pathology using the same set of specimens, the QCIGISH diagnostic grading model demonstrated higher AUC values for both best-case (BCC, indeterminate results considered as positive) and worst-case (WCC, indeterminate results considered as negative) conditions (0.99 vs 0.94 and 0.92 with *p* = 0.033 and *p* < 0.001, respectively, Additional file 1: Fig. S12 and Additional file 2: Table S14). QCIGISH demonstrated better accuracy than cytology and small biopsy pathology particularly for very early cancer stages (carcinoma in situ to Stage IB) (*p* = 0.041 for BCC and *p* = 0.004 for WCC, Fig. 5A and Additional file 2: Table S15). For carcinoma in situ and stage IA lung cancers, QCIGISH showed higher sensitivity of 96.0% (24/25, 95% CI 88.3–100.0%), as compared to standard pathological examination (68.0% = 17/25, 95% CI 49.7–86.3%). QCIGISH also demonstrated better accuracy for stage IB cancers [100.0% sensitivity (10/10) as compared to 70.0% sensitivity (7/10, 95% CI 41.6–98.4%)]. In addition, QCIGISH was consistently sensitive across late to terminal cancers (stages II to IV) (Fig. 5A and Additional file 2: Table S15). For SCLC, QCIGISH also showed 100.0% sensitivities to both limited stage and extensive stage (Fig. 5B and Additional file 2: Table S15).

The QCIGISH diagnostic grading model also equally demonstrated excellent accuracy for small pulmonary nodules (≤ 3 cm). For nodules smaller than 2 cm, QCIGISH’s sensitivity was noted at 100.0%, and specificity at 91.7% (95% CI 76.0–100.0%) (Fig. 5C, D, Additional file 2: Table S16 and S17). QCIGISH was also more accurate in diagnosing nodules with diameters from 2 to 3 cm with 100.0% sensitivity and 100.0% specificity (Fig. 5C, D, Additional file 2: Table S16 and S17). For all these measurements, QCIGISH was generally more sensitive [100.0% for < 2 cm (significant at *p* = 0.023 for WCC) and 100.0% for 2–3 cm (marginally significant at *p* = 0.134 for WCC)] than cytology and small biopsy pathology (66.7% for < 2 cm and 87.5% for 2–3 cm), although their specificities were relatively comparable (Fig. 5C, D, Additional file 2: Table S16 and S17).

To illustrate, QCIGISH was able to accurately classify two preoperatively diagnosed benign cases from small biopsy pathology (normal and benign lung tissues from bronchial biopsy) into malignant cases which were surgically verified as adenocarcinoma in situ and invasive adenocarcinoma (Fig. 5E).

## Discussion

The accurate diagnostic evaluation of pulmonary nodules and early-stage lung cancers currently remain a huge clinical challenge for standard diagnostic biopsies due to the insufficient tumor morphological evidence required to make a definitive cancer diagnosis. Epigenetic pathways have been and continue to remain a research hotspot in early lung cancer detection because of clearer evidence of their alterations in lung cancer carcinogenesis that most often predate malignant morphological changes [8]. Although epigenetic alterations have been recognized as potentially powerful tool for earlier diagnosis of lung cancer, epigenetic biomarkers have not been widely used in clinical practice. Altered genomic imprinting triggered by epigenetic changes is proposed to occur before tumor formation and promote tumor progression [8, 13]. While many researchers are exploring the changes of allele-specific DNA methylation in cancers, we focused on the transcriptional activity of imprinted gene loci on alleles, which could be better clinically visualized and quantified. In our previous study, we developed a novel QCIGISH method to evaluate the allelic expression status of imprinted genes and demonstrated the diagnostic significance of the elevated allelic expressions for imprinted genes as effective translational biomarkers for multiple cancers [31, 32].

In this study, we have developed a diagnostic grading model from highly sensitive and specific epigenetic imprinting-based biomarkers using the *GNAS*, *GRB10*, *SNRPN* and *HM13* gene panel that can be used as more accurate and definitive diagnostic biopsy evaluation of lung lesions. Our QCIGISH diagnostic grading model developed from the multiallelic imprinting alterations of this gene panel achieved excellent overall accuracy (99.1% sensitivity and 92.1% specificity) for diagnosing lung lesions from lung cytology and small biopsy specimens. In comparison with standard diagnostic biopsies, QCIGISH was more sensitive in detecting malignancies at their early curative stages (96.0% vs 68.0% sensitivity for CIS-Stage IA, 100.0% vs 68.6% sensitivity for Stage IB), and more accurate in distinguishing benign from malignant pulmonary nodules (100.0% vs 66.7% for < 2 cm, 100.0% vs 87.5% for 2–3 cm) with comparable specificities. All these findings demonstrated the epigenetic imprinting biomarker’s capability to effectively provide clearer and more advanced evidence of cancer than morphology. These excellent diagnostic performance and predictive ability of QCIGISH make this molecular test a robust and useful clinical decision-enabling technology which could improve the accuracy for standard diagnostic biopsies particularly for early-stage lung cancers and small pulmonary nodules.

LOI of the *GNAS* gene has been reported to be associated with increased risks of multiple cancers including thyroid cancer, skin cancer, osteosarcoma and neurofibromatosis [33]. Similarly, studies have shown the relationship between the aberrant methylation of the *GRB10* gene and invasive breast cancer [34]. Hypomethylation for the *SNRPN* gene has also been linked to breast cancer and seminoma [35, 36]. Moreover, research has shown that LOI and upregulation for the *HM13* gene have both been involved with breast cancer, in addition to its functional relationship with glioblastoma progression [14, 37]. However, to our knowledge, the potential relationship between these four imprinted genes toward lung cancer development has yet to be explored. Using this four-gene panel, our QCIGISH method has detected significantly elevated BAE, MAE and TE in lung cancers as compared to benign lesions. Increased allelic expression can result from either LOI with the normally silenced copy of the gene reactivated [13] or copy number variation (CNV) with the active copy of the gene amplified but the inactive copy still silenced [19]. Our QCIGISH method only detects the transcriptionally active copies which potentially limits the capability to determine the specific mechanisms driving the increased allelic expression. Further studies are needed to investigate and explore the prospective roles of LOI and CNV in the increased allelic expressions of imprinted genes during lung cancer development. Our additional analyses across the different disease subtypes identified MAE as the more effective malignancy biomarker over BAE and TE. This observation might be particularly related to the precocious occurrence of imprinting alterations in tumors. Higher TE was reported for both lung inflammatory lesions and lung cancer and therefore determined as not optimally effective in differentiating malignancy. Higher BAE, representing early epigenetic or genetic alterations of imprinted genes which might precede morphological changes in cells and tissues indicative of malignancy, demonstrated unsatisfactory diagnostic specificity for lung diseases. Higher MAE, which subsequently develops after BAE, effectively demonstrated good malignancy discrimination consistent with current pathological evidence. Further exploratory studies are, however, needed to further investigate the biological implications of elevated TE, BAE and MAE levels toward other cancer types with varying pathophysiology.

From the simultaneous comparative pathological evaluation performed using QCIGISH and H&E staining on the same block resected near the cancer-bearing tissue region, elevated allelic expressions effectively conformed with malignant morphological features. These results highlighted the diagnostic significance of epigenetic imprinting alterations as clear and reliable distinguishing markers for lung malignancy. Therefore, epigenetic imprinting biomarkers could effectively provide a definitive diagnosis of lung cancers especially when clear tumor morphological evidence is insufficient.

Clinical studies have shown that nodule morphological characteristics such as diameter size, among others, have been associated with an increased risk of malignancy [38]. However, current preoperative biopsies for these small nodules may be inadequate to make a definitive diagnosis. While diagnostic guidelines differ between countries, nodules with diameters smaller than 2 cm are generally recommended for a 24-month CT follow-up instead of immediate surgical intervention. Therefore, progressive malignant tumors are not promptly identified to permit timely clinical management [39]. In recent years, as more sub-centimeter nodules are detected with the expanding population receiving LDCT screening [3], more accurate and definitive diagnostic methods for pulmonary nodules have become increasingly essential.

As more than 50% of CT-detected lung cancers are reported as Stage I [3], QCIGISH addresses this unmet clinical need of accurately detecting potentially malignant cases among small pulmonary nodules which are usually at their early stages. Our results showed that QCIGISH could positively detect truly malignant cases from biopsies potentially diagnosed as benign or indeterminate due to unclear morphological evidence, helping tackle a significant clinical diagnostic challenge [40]. The application of QCIGISH now enables the discovery of these early lung cancers which could lead to better clinical outcomes by permitting timely treatment and reducing the uncertainty of delayed monitoring of malignant cases, ultimately increasing patients’ survival.

It is interesting that the diagnostic grading model developed from NSCLC samples can also be applied for SCLC, as we discovered that both shared similar epigenetic alterations using the imprinted gene panel despite their different cell origins and distinct genetic alterations [41]. As SCLC patients have very poor prognosis because of late-stage diagnosis [42], QCIGISH could be clinically useful by also effectively supporting the early prediction and accurate diagnosis of SCLC using cytology and small biopsy specimens.

This study had several limitations. First, our validation cohort consisted of only six Chinese hospitals—a more conclusive validation could be achieved using a prospective large-scale evaluation involving more medical centers and higher patient case numbers with more diverse clinical characteristics and disease subtypes; second, we monitored the clinically diagnosed benign cases for only two years—a longer follow-up period could provide a more accurate clinical validation especially for slowly progressive lung cancer cases; third, there are opportunities to further optimize the gene probes that we used—more exploration could be proceeded to additionally improve the diagnostic model’s accuracy and better characterize more cancer subtypes while maintaining a minimally efficient number of probes; and lastly, due to a substantial number of cases with unclear LDCT features obtained particularly for benign lesions, radiological features such as solid, subsolid and ground glass were not considered in the analysis although their inclusion could have provided vital perspectives toward malignancy differentiation especially for early-stage lung cancers.

## Conclusions

This study demonstrated how epigenetic imprinting biomarkers effectively provided clearer and more advanced differentiation of lung cancer than morphology. The high sensitivity and specificity make this test particularly effective in ruling-out and ruling-in malignancy in lung lesions. Capitalizing on the strength of highly sensitive and specific epigenetic translational biomarkers and a clinically viable technique, QCIGISH represents a reliable epigenetics-based approach and a decision-enabling technology for a more accurate and definitive cytology and small biopsy specimen diagnosis of small pulmonary nodules and early-stage lung cancers. Thus, as an adjunctive procedure to standard biopsies for lung lesions, this novel imprinting biomarker-based diagnostic test has a high potential to improve current clinical treatment decisions, and ultimately health outcomes.

## Methods

### Study design and sample collection

A total of 431 subjects recruited from eight Chinese medical centers were found eligible for the study and were divided into three sets based on specimen type and sample collection date as shown in Fig. 1 and Additional file 1: Figure S1. For the imprinted gene screening, biomarker pre-selection and diagnostic model building set, 283 formalin-fixed and paraffin-embedded (FFPE) surgically resected and histologically diagnosed lung tissue specimens were retrospectively collected. For the model testing set, 35 bronchoscopy and transthoracic CNB sampled lung small biopsy specimens were retrospectively collected. For the blinded model validation set, 240 patients with lung lesions detected on chest CT scans (see Additional file 2: Materials and Methods) were recruited and were clinically examined using bronchoscopy or transthoracic CNB (see Additional file 2: Materials and Methods). The sources and collection time periods of the samples are shown in Fig. 1 and Additional file 1: Figure S2. The demographic and clinical characteristics of the study subjects are provided in Table 1 and Additional file 2: Table S1. The corresponding surgical histopathology was reviewed by three pathologists, namely RS, HY and WH. CB maintained the blinded data and oversaw the evaluation process. This study has been registered in clinicaltrials.gov (clinical trial ID: NCT03882684).

### Sample preparation and QCIGISH detection

The lung tissue specimens and the lung cytology and small biopsy specimens were prepared using a previously described procedure [30]. Briefly, FFPE tissue samples were cut into 10-μm sections and mounted on positively charged slides. Cytology and small biopsy samples were fixed immediately after sampling in 10% NBF (neutral buffered formalin) for 48 h at RT. The dissociated cells were directly mounted onto positively charged slides. With probes targeting the non-coding intronic regions of nascent RNAs for the *GNAS*, *GRB10*, *SNRPN* and *HM13* imprinted genes, ISH was applied following a previously described procedure using RNAscope 2.5 HD Assay kit (Advanced Cell Diagnostics, Newark, CA, USA) [30]. The detected gene-expressing sites were visualized as distinct red or brown dots under common bright field microscope after signal amplification (Fig. 2A). The numbers of nuclei containing no signal (N_0_), one signal (N_1_), two signals (N_2_), and more than two signals (N_2+_) were collected from the microscopic images using the procedure as previously described [30] and were used to calculate the respective biallelic expression (BAE), multiallelic expression (MAE) and total expression (TE) according to the equations shown in Fig. 2A. The minimum nuclei count applied for processing tissue and cell samples using QCIGISH was determined as 1500 and 1000, respectively, with details described under Additional file 2: Materials and Methods (Additional file 1: Fig. S13). The technicians who performed QCIGISH detection have no pathology background and were blinded to the simultaneous H&E staining results.

### Imprinted gene screening and biomarker pre-selection

In our previous study, we have identified three imprinted genes *GNAS*, *GRB10* and *SNRPN* for the diagnosis of ten cancer types including lung cancer [30]. Aiming to potentially improve the diagnostic performance of the QCIGISH binary classification model with 92% sensitivity and 88% specificity [30], we evaluated a new imprinted gene *HM13* which was reported to be involved in breast cancer and glioblastoma [14, 37].

To subsequently evaluate the imprinting alteration signatures and pre-select candidate biomarkers for diagnostic model building, the discrimination performance for the BAE, MAE and TE measurements for each imprinted gene was individually assessed in a pooled analysis for each disease subtype (see Additional file 2: Materials and Methods, Additional file 1: Fig. S5).

### Diagnostic grading model building and testing

Using the imprinting patterns from the most effective markers determined in the prior analysis, the previously developed malignancy classification model structure using an ensemble of individual gene classifiers [30] was updated by extending the diagnostic output from binary response to a five-level grading system (Additional file 1: Fig. S6 and Fig. S7). The development of the diagnostic algorithm and the corresponding evaluation and optimization of the model thresholds in the model building set are detailed under Additional file 2: Materials and Methods. Based on the evaluation results, a number of models with varying threshold combinations which demonstrated an optimal range of diagnostic accuracies were further tested in an independent set of 30 cytology and small biopsy specimens. The candidate model which showed the best diagnostic performance after testing was determined as the final model, with all threshold specifications locked prior to validation in an independent cohort of 155 patients.

### Statistics

Continuous variables were reported as medians with interquartile ranges (IQR), while frequencies and proportions were reported for categorical variables. Continuous clinical variables were compared between groups using the Mann–Whitney U and Kruskal–Wallis tests, as applicable, driven by the non-normal distributions determined using the Shapiro–Wilk test [43]. Dunn’s test was performed as a post hoc test for the pairwise comparisons between each independent group with Bonferroni correction applied during *p*-value determination [44]. Categorical clinical variables were compared using Chi-square or Fisher exact tests, as applicable.

Diagnostic discrimination performance was assessed and compared using the receiver operating characteristics area under the curve (ROC AUC) metric with 95% confidence intervals determined using the DeLong method [45]. Sensitivity, specificity and their respective normal-based 95% confidence intervals were computed using standard methods. Diagnostic sensitivities and specificities obtained using QCIGISH were evaluated against cytology and small biopsy pathology using McNemar’s test for paired data [46].

All hypothesis tests were done in a two-sided manner, with computed *p* < 0.05 considered to be statistically significant. All statistical analyses and visualizations were performed using R software (version 3.5.0) [47]. Sample size justification is described under Additional file 2.

## Supplementary Information

**Additional file 1**. Supplementary Figures.

**Additional file 2**. Supplementary Materials, Methods and Tables.

## Data Availability

The datasets used and/or analyzed during the current study are available from Dr. Ning Zhou (E-mail: zhou.ning@lisenid.com) on reasonable request.

## References

[1] Siegel RL, Miller KD, Jemal A (2019). Cancer statistics, 2019. CA: Cancer J Clin.

[2] Goldstraw P, Crowley J, Chansky K, Giroux DJ, Groome PA, Rami-Porta R, Postmus PE, Rusch V, Sobin L, International Association for the Study of Lung Cancer International Staging C, et al. The IASLC lung cancer staging project: proposals for the revision of the TNM stage groupings in the forthcoming (seventh) edition of the TNM Classification of malignant tumours. J Thorac Oncol. 2007; 2(8):706–714. 10.1097/JTO.0b013e31812f3c1a 17762336

[3] National Lung Screening Trial Research T, Aberle DR, Adams AM, Berg CD, Black WC, Clapp JD, Fagerstrom RM, Gareen IF, Gatsonis C, Marcus PM, et al. Reduced lung-cancer mortality with low-dose computed tomographic screening. N Engl J Med 2011; 365(5):395–409. 10.1056/NEJMoa1102873 PMC4356534 21714641

[4] Rivera MP, Mehta AC, Wahidi MM (2013). Establishing the diagnosis of lung cancer: diagnosis and management of lung cancer, 3rd ed: American College of Chest Physicians evidence-based clinical practice guidelines. Chest.

[5] Tarro G, Perna A, Esposito C (2005). Early diagnosis of lung cancer by detection of tumor liberated protein. J Cell Physiol.

[6] Aravanis AM, Lee M, Klausner RD (2017). Next-generation sequencing of circulating tumor DNA for early cancer detection. Cell.

[7] Kruglyak KM, Lin E, Ong FS (2016). Next-generation sequencing and applications to the diagnosis and treatment of lung cancer. Adv Expert Med Biol.

[8] Feinberg AP (2018). The key role of epigenetics in human disease prevention and mitigation. N Engl J Med.

[9] Fleischhacker M, Dietrich D, Liebenberg V, Field JK, Schmidt B (2013). The role of DNA methylation as biomarkers in the clinical management of lung cancer. Expert Rev Respir Med.

[10] Wang YW, Ma X, Zhang YA, Wang MJ, Yatabe Y, Lam S, Girard L, Chen JY, Gazdar AF (2016). ITPKA gene body methylation regulates gene expression and serves as an early diagnostic marker in lung and other cancers. J Thorac Oncol.

[11] Hassanein M, Callison JC, Callaway-Lane C, Aldrich MC, Grogan EL, Massion PP (2012). The state of molecular biomarkers for the early detection of lung cancer. Cancer Prevent Res.

[12] Barlow DP, Bartolomei MS (2014). Genomic imprinting in mammals. Cold Spring Harbor Perspect Biol.

[13] Jelinic P, Shaw P (2007). Loss of imprinting and cancer. J Pathol: A J Pathol Soc G B Irel.

[14] Goovaerts T, Steyaert S, Vandenbussche CA, Galle J, Thas O, Van Criekinge W, De Meyer T (2018). A comprehensive overview of genomic imprinting in breast and its deregulation in cancer. Nat Commun.

[15] Holm TM, Jackson-Grusby L, Brambrink T, Yamada Y, Rideout WM, Jaenisch R (2005). Global loss of imprinting leads to widespread tumorigenesis in adult mice. Cancer Cell.

[16] Kohda M, Hoshiya H, Katoh M, Tanaka I, Masuda R, Takemura T, Fujiwara M, Oshimura M (2001). Frequent loss of imprinting of IGF2 and MEST in lung adenocarcinoma. Mol Carcinogen.

[17] Monk D, Mackay DJG, Eggermann T, Maher ER, Riccio A (2019). Genomic imprinting disorders: lessons on how genome, epigenome and environment interact. Nat Rev Genet.

[18] Enterina JR, Enfield KSS, Anderson C, Marshall EA, Ng KW, Lam WL (2017). DLK1-DIO3 imprinted locus deregulation in development, respiratory disease, and cancer. Expert Rev Respir Med.

[19] Martin-Trujillo A, Vidal E, Monteagudo-Sanchez A, Sanchez-Delgado M, Moran S, Hernandez Mora JR, Heyn H, Guitart M, Esteller M, Monk D (2017). Copy number rather than epigenetic alterations are the major dictator of imprinted methylation in tumors. Nat Commun.

[20] Wissink EM, Vihervaara A, Tippens ND, Lis JT (2019). Nascent RNA analyses: tracking transcription and its regulation. Nat Re Genet.

[21] Lim B (2018). Imaging transcriptional dynamics. Curr Opin Biotechnol.

[22] Soler M, Boque-Sastre R, Guil S (2017). RNA-FISH to study regulatory RNA at the site of transcription. Methods Mol Biol.

[23] Brown JM, Buckle VJ (2010). Detection of nascent RNA transcripts by fluorescence in situ hybridization. Methods Mol Biol.

[24] Jouvenot Y, Poirier F, Jami J, Paldi A (1999). Biallelic transcription of Igf2 and H19 in individual cells suggests a post-transcriptional contribution to genomic imprinting. Curr Biol.

[25] Ohno M, Aoki N, Sasaki H (2001). Allele-specific detection of nascent transcripts by fluorescence in situ hybridization reveals temporal and culture-induced changes in Igf2 imprinting during pre-implantation mouse development. Genes Cells.

[26] Lahbib-Mansais Y, Barasc H, Marti-Marimon M, Mompart F, Iannuccelli E, Robelin D, Riquet J, Yerle-Bouissou M (2016). Expressed alleles of imprinted IGF2, DLK1 and MEG3 colocalize in 3D-preserved nuclei of porcine fetal cells. BMC Cell Biol.

[27] Kohda A, Taguchi H, Okumura K (2001). Visualization of biallelic expression of the imprinted SNRPN gene induced by inhibitors of DNA methylation and histone deacetylation. Biosci Biotechnol Biochem.

[28] Borensztein M, Okamoto I, Syx L, Guilbaud G, Picard C, Ancelin K, Galupa R, Diabangouaya P, Servant N, Barillot E (2017). Contribution of epigenetic landscapes and transcription factors to X-chromosome reactivation in the inner cell mass. Nat Commun.

[29] Borensztein M (2021). Investigating the inner cell mass of the mouse blastocyst by combined immunofluorescence staining and RNA fluorescence in situ hybridization. Methods Mol Biol.

[30] Shen R, Cheng T, Xu C, Yung RC, Bao J, Li X, Yu H, Lu S, Xu H, Wu H (2020). Novel visualized quantitative epigenetic imprinted gene biomarkers diagnose the malignancy of ten cancer types. Clin Epigenet.

[31] Yung R, Cheng T, Li X, Wang X, Si H, Zhao P, Shen R, Zhou J, Yu H, Ding M (2019). P.109-12 in-situ hybridization visual scoring of epigenetic imprinting genes improves early diagnosis and grading of lung cancers. J Thoracic Oncol.

[32] Shen R, Wu HX, Cheng T, Li X, Wang X, Si H, Liu Y, Yin M, Yang S, Zhang Y (2019). Epigenetic panel of imprinted genes can enhance the accuracy of FNA cytopathology for thyroid cancer. Thyroid.

[33] Murrell A (2006). Genomic imprinting and cancer: from primordial germ cells to somatic cells. ScientificWorldJournal.

[34] Barrow TM, Barault L, Ellsworth RE, Harris HR, Binder AM, Valente AL, Shriver CD, Michels KB (2015). Aberrant methylation of imprinted genes is associated with negative hormone receptor status in invasive breast cancer. Int J Cancer.

[35] Harrison K, Hoad G, Scott P, Simpson L, Horgan GW, Smyth E, Heys SD, Haggarty P (2015). Breast cancer risk and imprinting methylation in blood. Clin Epigenetics.

[36] Uribe-Lewis S, Woodfine K, Stojic L, Murrell A (2011). Molecular mechanisms of genomic imprinting and clinical implications for cancer. Expert Rev Mol Med.

[37] Wei JW, Cai JQ, Fang C, Tan YL, Huang K, Yang C, Chen Q, Jiang CL, Kang CS (2017). Signal peptide peptidase, encoded by HM13, contributes to tumor progression by affecting EGFRvIII secretion profiles in glioblastoma. CNS Neurosci Ther.

[38] McWilliams A, Tammemagi MC, Mayo JR, Roberts H, Liu G, Soghrati K, Yasufuku K, Martel S, Laberge F, Gingras M (2013). Probability of cancer in pulmonary nodules detected on first screening CT. N Engl J Med.

[39] Wah W, Stirling RG, Ahern S, Earnest A (2020). Influence of timeliness and receipt of first treatment on geographic variation in non-small cell lung cancer mortality. Int J Cancer.

[40] Butnor KJ (2008). Avoiding underdiagnosis, overdiagnosis, and misdiagnosis of lung carcinoma. Arch Pathol Lab Med.

[41] Rodriguez-Canales J, Parra-Cuentas E, Wistuba II (2016). Diagnosis and molecular classification of lung cancer. Cancer Treat Res.

[42] van Meerbeeck JP, Fennell DA, De Ruysscher DK (2011). Small-cell lung cancer. Lancet.

[43] Kruskal WH, Wallis WA (1952). Use of ranks in one-criterion variance analysis. J Am Stat Assoc.

[44] Dunn OJ (1964). Multiple comparisons using rank sums. Technometrics.

[45] DeLong ER, DeLong DM, Clarke-Pearson DL (1988). Comparing the areas under two or more correlated receiver operating characteristic curves: a nonparametric approach. Biometrics.

[46] Mc NQ (1947). Note on the sampling error of the difference between correlated proportions or percentages. Psychometrika.

[47] Chan BKC (2018). Applied statistics for human genetics using R. Adv Exp Med Biol.

