# Supplementary material for: Epigenetic imprinting alterations as effective diagnostic biomarkers for early-stage lung cancer and small pulmonary nodules
Source: Clin Epigenetics. 2021 Dec 14;13:220. doi: 10.1186/s13148-021-01203-5 (PMC8672623; doi:10.1186/s13148-021-01203-5)
Supplement: Supplementary file 1 — Additional file 1. Supplementary Figures. [file 13148_2021_1203_MOESM1_ESM.pdf]

Fig. S1

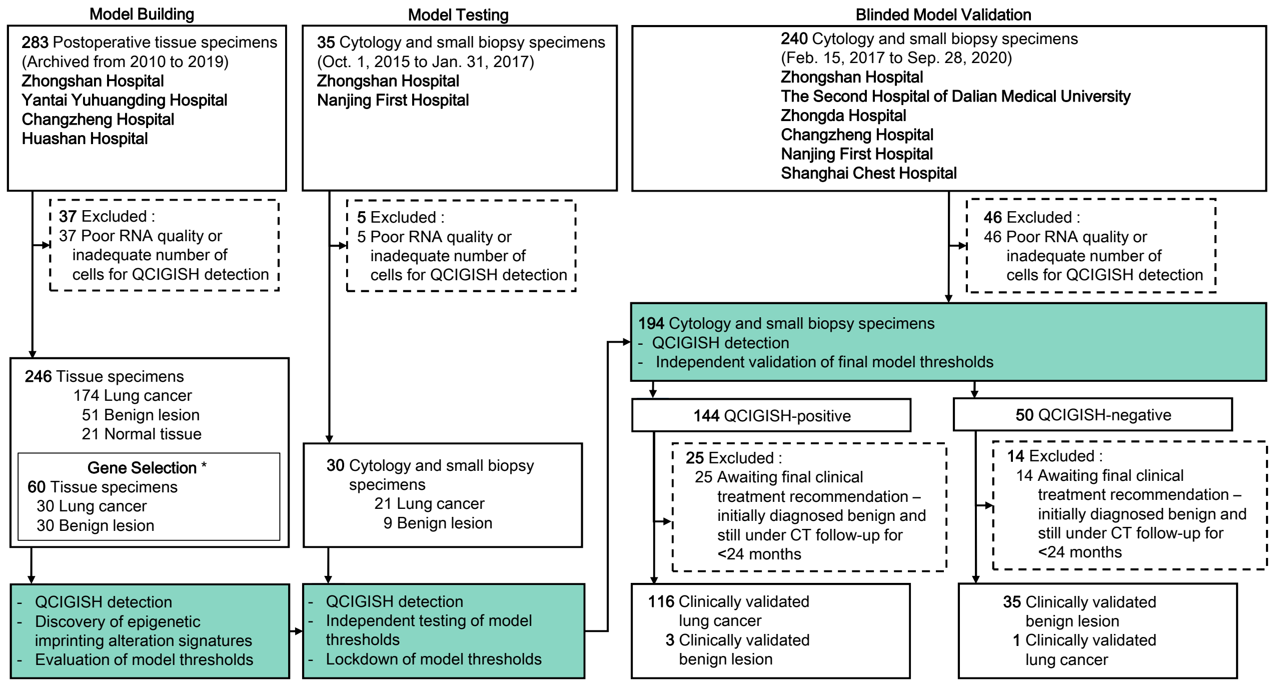

Model Building
283 Postoperative tissue specimens
(Archived from 2010 to 2019)
Zhongshan Hospital
Yantai Yuhuangding Hospital
Changzheng Hospital
Huashan Hospital
37 Excluded :
37 Poor RNA quality or inadequate number of cells for QCIGISH detection
246 Tissue specimens
174 Lung cancer
51 Benign lesion
21 Normal tissue
Gene Selection *
60 Tissue specimens
30 Lung cancer
30 Benign lesion
- QCIGISH detection
- Discovery of epigenetic imprinting alteration signatures
- Evaluation of model thresholds
Model Testing
35 Cytology and small biopsy specimens
(Oct. 1, 2015 to Jan. 31, 2017)
Zhongshan Hospital
Nanjing First Hospital
5 Excluded :
5 Poor RNA quality or inadequate number of cells for QCIGISH detection
30 Cytology and small biopsy specimens
21 Lung cancer
9 Benign lesion
- QCIGISH detection
- Independent testing of model thresholds
- Lockdown of model thresholds
Blinded Model Validation
240 Cytology and small biopsy specimens
(Feb. 15, 2017 to Sep. 28, 2020)
Zhongshan Hospital
The Second Hospital of Dalian Medical University
Zhongda Hospital
Changzheng Hospital
Nanjing First Hospital
Shanghai Chest Hospital
46 Excluded :
46 Poor RNA quality or inadequate number of cells for QCIGISH detection
194 Cytology and small biopsy specimens
- QCIGISH detection
- Independent validation of final model thresholds
144 QCIGISH-positive
25 Excluded :
25 Awaiting final clinical treatment recommendation – initially diagnosed benign and still under CT follow-up for <24 months
116 Clinically validated lung cancer
3 Clinically validated benign lesion
50 QCIGISH-negative
14 Excluded :
14 Awaiting final clinical treatment recommendation – initially diagnosed benign and still under CT follow-up for <24 months
35 Clinically validated benign lesion
1 Clinically validated lung cancer

Fig. S2

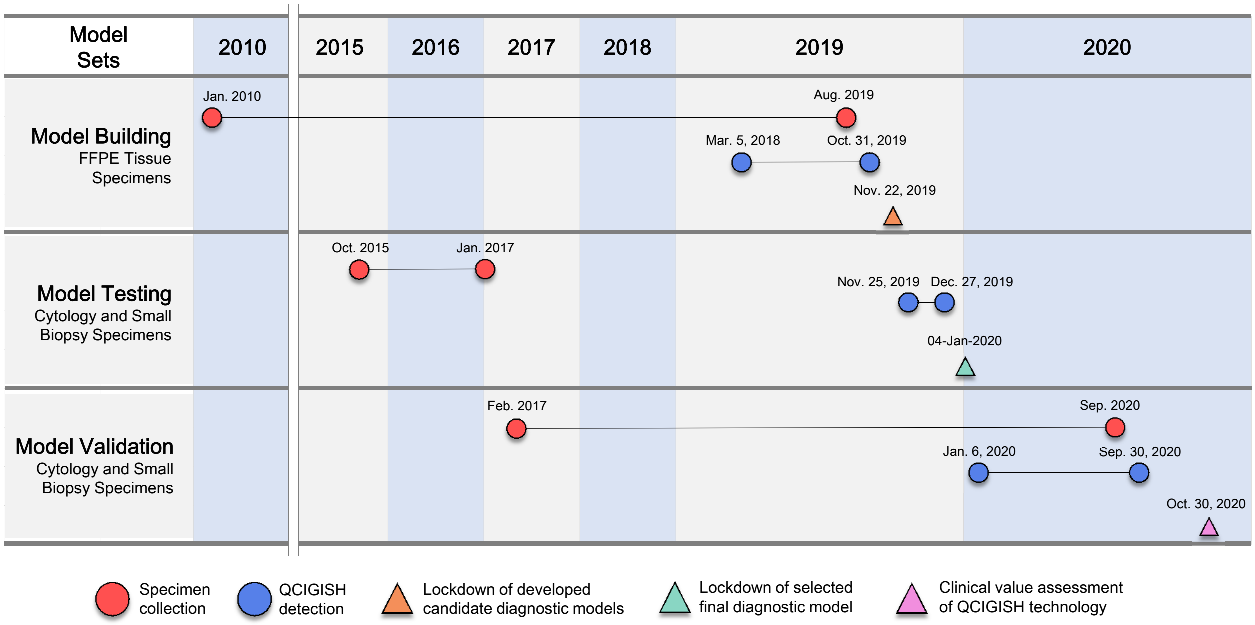

Model Sets
2010
2015
2016
2017
2018
2019
2020
Model Building
FFPE Tissue Specimens
Jan. 2010
Aug. 2019
Mar. 5, 2018
Oct. 31, 2019
Nov. 22, 2019
Model Testing
Cytology and Small Biopsy Specimens
Oct. 2015
Jan. 2017
Nov. 25, 2019
Dec. 27, 2019
04-Jan-2020
Model Validation
Cytology and Small Biopsy Specimens
Feb. 2017
Sep. 2020
Jan. 6, 2020
Sep. 30, 2020
Oct. 30, 2020
Specimen collection
QCIGISH detection
Lockdown of developed candidate diagnostic models
Lockdown of selected final diagnostic model
Clinical value assessment of QCIGISH technology

Fig. S3

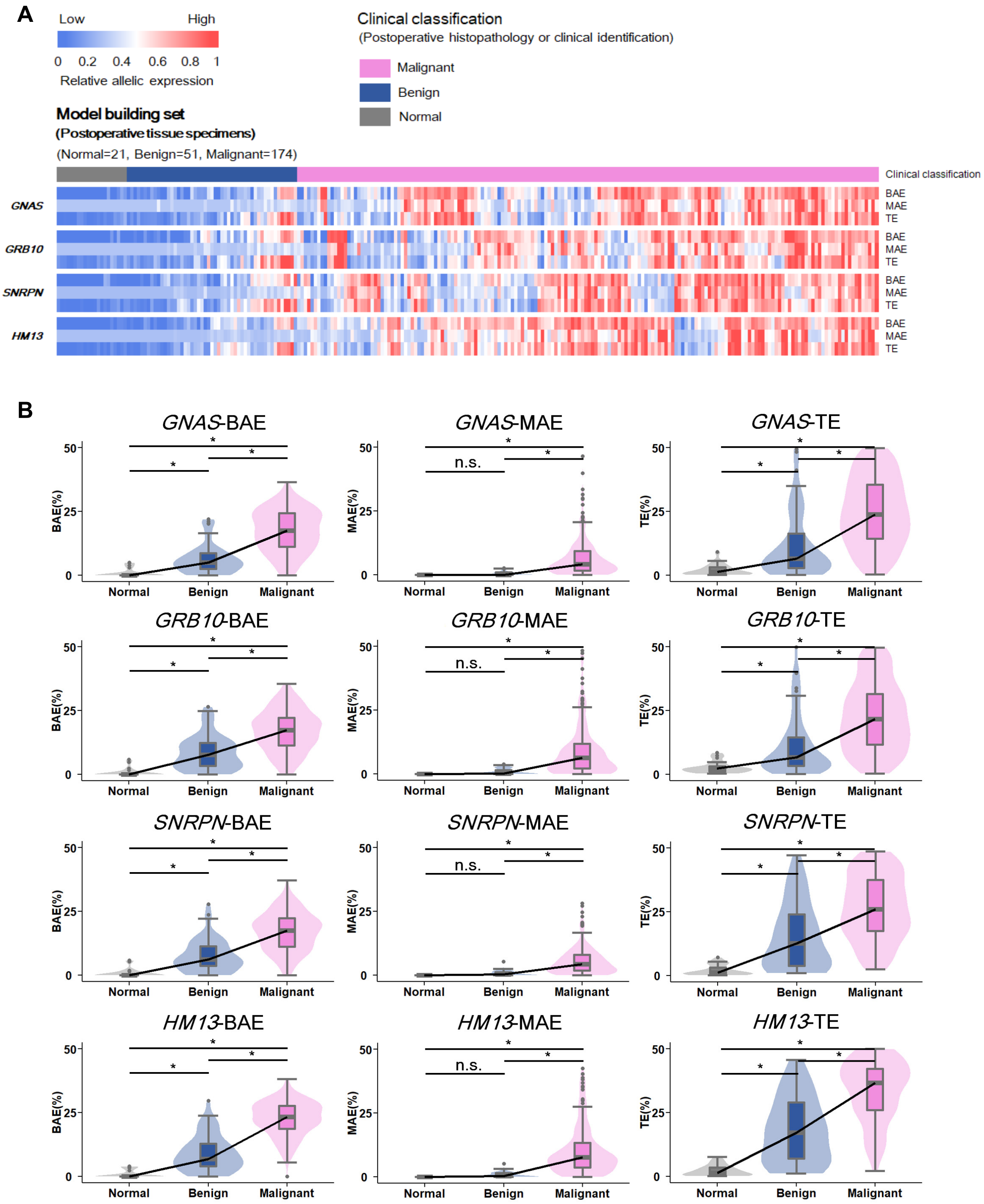

A
Low
High
0 0.2 0.4 0.6 0.8 1
Relative allelic expression
Model building set
(Postoperative tissue specimens)
(Normal=21, Benign=51, Malignant=174)
Clinical classification
(Postoperative histopathology or clinical identification)
Malignant
Benign
Normal
Clinical classification
GNAS
GRB10
SNRPN
HM13
BAE
MAE
TE
B
GNAS-BAE
GNAS-MAE
GNAS-TE
GRB10-BAE
GRB10-MAE
GRB10-TE
SNRPN-BAE
SNRPN-MAE
SNRPN-TE
HM13-BAE
HM13-MAE
HM13-TE
BAE(%)
MAE(%)
TE(%)
n.s.
Normal
Benign
Malignant

Fig. S4

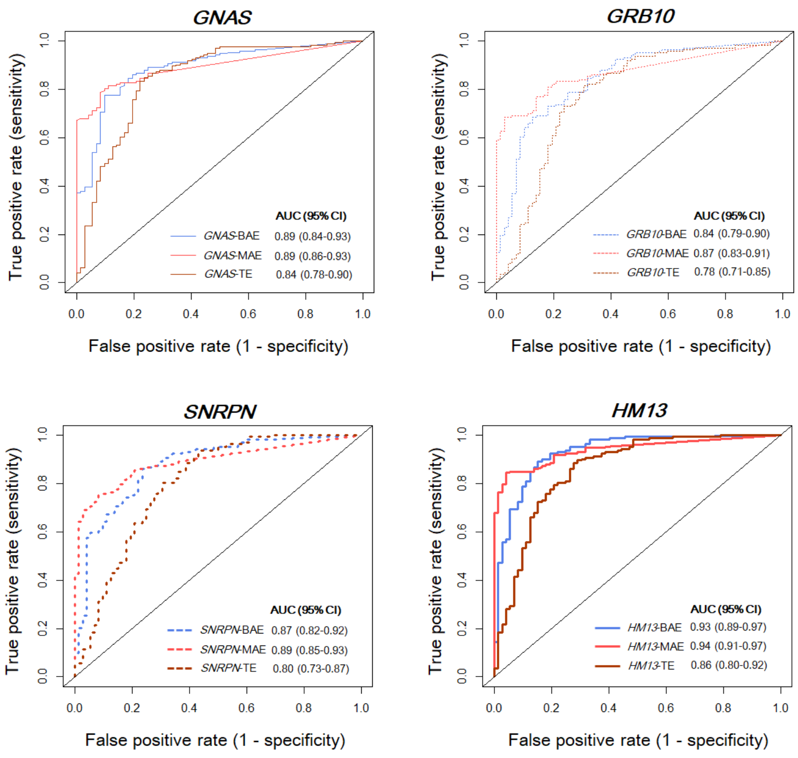

GNAS
AUC (95% CI)
GNAS-BAE 0.89 (0.84-0.93)
GNAS-MAE 0.89 (0.86-0.93)
GNAS-TE 0.84 (0.78-0.90)
GRB10
AUC (95% CI)
GRB10-BAE 0.84 (0.79-0.90)
GRB10-MAE 0.87 (0.83-0.91)
GRB10-TE 0.78 (0.71-0.85)
SNRPN
AUC (95% CI)
SNRPN-BAE 0.87 (0.82-0.92)
SNRPN-MAE 0.89 (0.85-0.93)
SNRPN-TE 0.80 (0.73-0.87)
HM13
AUC (95% CI)
HM13-BAE 0.93 (0.89-0.97)
HM13-MAE 0.94 (0.91-0.97)
HM13-TE 0.86 (0.80-0.92)
True positive rate (sensitivity)
False positive rate (1 - specificity)
0.0
0.2
0.4
0.6
0.8
1.0

Fig. S5

Highly specific imprinted gene biomarker*

Highly sensitive imprinted gene biomarker*

Highly accurate imprinted gene biomarker*

* Sensitivity, specificity or overall accuracy >= 80%

** Best biomarker for each disease subtype

		Individual gene BAE, MAE and TE classification accuracy for lung normal, benign and malignant subtypes using maximum (sensitivity + specificity)											
**Accuracy by clinical classification subtypes**	**n**	***GNAS*-BAE**	***GNAS*-MAE**	***GNAS*-TE**	***GRB10*-BAE**	***GRB10*-MAE**	***GRB10*-TE**	***SNRPN*-BAE**	***SNRPN*-MAE**	***SNRPN*-TE**	***HM13*-BAE**	***HM13*-MAE**	***HM13*-TE**
**Specificity for normal and benign**	**72**	**90.28% ****	**90.28%**	**76.39%**	**87.50%**	**97.22% ****	**69.44%**	**76.39%**	**91.67%**	**56.94%**	**84.72%**	**95.83%**	**72.22%**
**Specificity for all normal**	**21**	**100.00% ****	**100.00% ****	**100.00% ****	**100.00% ****	**100.00% ****	**100.00% ****	**100.00% ****	**100.00% ****	**100.00% ****	**100.00% ****	**100.00% ****	**100.00% ****
Specificity for normal	21	100.00% **	100.00% **	100.00% **	100.00% **	100.00% **	100.00% **	100.00% **	100.00% **	100.00% **	100.00% **	100.00% **	100.00% **
**Specificity for all benign**	**51**	**86.27% ****	**86.27%**	**66.67%**	**82.35%**	**96.08% ****	**56.86%**	**66.67%**	**88.24%**	**39.22%**	**78.43%**	**94.12%**	**60.78%**
Specificity for pulmonary sclerosing pneumocytoma	13	92.31% **	92.31%	84.62% **	92.31% **	100.00% **	76.92%	69.23%	92.31%	23.08%	84.62%	92.31%	69.23%
Specificity for cryptogenic organizing pneumonia	10	90.00% **	100.00% **	40.00%	80.00%	100.00% **	40.00%	60.00%	90.00%	10.00%	80.00%	100.00% **	50.00%
Specificity for pulmonary cryptococcosis	10	90.00% **	80.00%	80.00% **	90.00% **	90.00%	80.00% **	90.00% **	90.00%	80.00% **	90.00% **	100.00% **	70.00%
Specificity for pulmonary tuberculosis	10	70.00%	80.00%	70.00%	70.00%	100.00% **	60.00%	70.00%	90.00%	60.00%	50.00%	80.00%	50.00%
Specificity for pulmonary inflammatory pseudotumor	3	100.00% **	100.00% **	100.00% **	66.67%	100.00% **	33.33%	66.67%	100.00% **	66.67%	66.67%	100.00% **	66.67%
Specificity for inflammation	3	66.67%	33.33%	33.33%	100.00% **	100.00% **	0.00%	0.00%	66.67%	0.00%	100.00% **	100.00% **	66.67%
Specificity for granuloma	2	100.00% **	100.00% **	0.00%	50.00%	50.00%	0.00%	50.00%	50.00%	0.00%	100.00% **	100.00% **	50.00%
**Sensitivity for all malignant**	**174**	**77.59%**	**80.46%**	**85.06%**	**68.39%**	**68.39%**	**81.61%**	**86.21%**	**75.86%**	**93.68% ****	**89.08% ****	**84.48% ****	**88.51%**
Sensitivity for adenocarcinoma	82	75.61%	79.27%	85.37%	70.73%	74.39%	82.93%	90.24% **	80.49%	93.90% **	85.37%	87.80% **	89.02%
Sensitivity for squamous cell carcinoma	76	78.95%	81.58%	85.53%	75.00%	73.68%	86.84%	78.95%	65.79%	92.11%	94.74% **	90.79% **	93.42% **
Sensitivity for adenocarcinoma *in situ*	12	83.33%	75.00%	83.33%	16.67%	0.00%	41.67%	100.00% **	100.00% **	100.00% **	75.00%	33.33%	58.33%
Sensitivity for adenosquamous cell carcinoma	2	50.00%	100.00% **	50.00%	0.00%	0.00%	50.00%	100.00% **	100.00% **	100.00% **	100.00% **	50.00%	100.00% **
Sensitivity for large cell carcinoma	1	100.00% **	100.00% **	100.00% **	100.00% **	100.00% **	100.00% **	100.00% **	100.00% **	100.00% **	100.00% **	0.00%	0.00%
Sensitivity for adenocarcinoma, squamous cell and neuroendocrine carcinoma	1	100.00% **	100.00% **	100.00% **	100.00% **	100.00% **	100.00% **	100.00% **	100.00% **	100.00% **	100.00% **	100.00% **	100.00% **
**Accuracy for all cases**	**246**	**81.30%**	**83.33%**	**82.52%**	**73.98%**	**76.83%**	**78.05%**	**83.33%**	**80.49%**	**82.93%**	**87.80% ****	**87.80% ****	**83.74% ****

Fig. S6

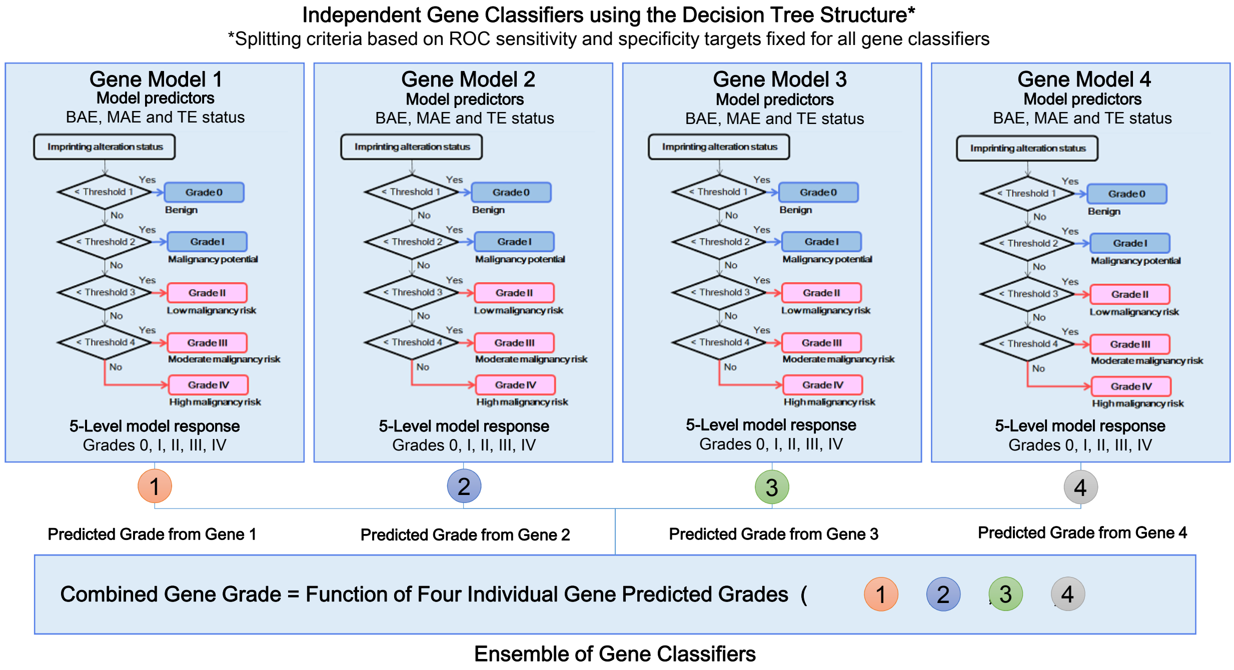

Independent Gene Classifiers using the Decision Tree Structure*
*Splitting criteria based on ROC sensitivity and specificity targets fixed for all gene classifiers
Gene Model 1
Model predictors
BAE, MAE and TE status
Imprinting alteration status
< Threshold 1
Yes
No
Grade 0
Benign
< Threshold 2
Grade I
Malignancy potential
< Threshold 3
Grade II
Low malignancy risk
< Threshold 4
Grade III
Moderate malignancy risk
Grade IV
High malignancy risk
5-Level model response
Grades 0, I, II, III, IV
Gene Model 2
Gene Model 3
Gene Model 4
1
2
3
4
Predicted Grade from Gene 1
Predicted Grade from Gene 2
Predicted Grade from Gene 3
Predicted Grade from Gene 4
Combined Gene Grade = Function of Four Individual Gene Predicted Grades (
Ensemble of Gene Classifiers

Fig. S7

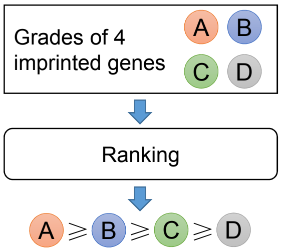

Combined gene model	Grades considered	Conditions	Combined gene grade
**Using the top 1 grade**	A	-	A
**Using the top 2 grades**	A B	If A = B<br>If A > B	A<br>A - 1**
**Using the top 3 grades***	A B C	If A = B<br>If A > B	A<br>A - 1**
**Using all 4 grades***	A B C D	If A = B<br>If A > B	A<br>A - 1**

A, B, C, D: Grades of each imprinted gene ranked from highest to lowest
* Only Grades A and B obtained from the two genes with the highest alterations are considered for the combined gene grade.
** When A > B, combined gene grade is equal to (A – 1) to maximize the predictive ability of the best gene, while minimally adjusting for potential variations in the predicted grades between the two best genes.

Fig. S8

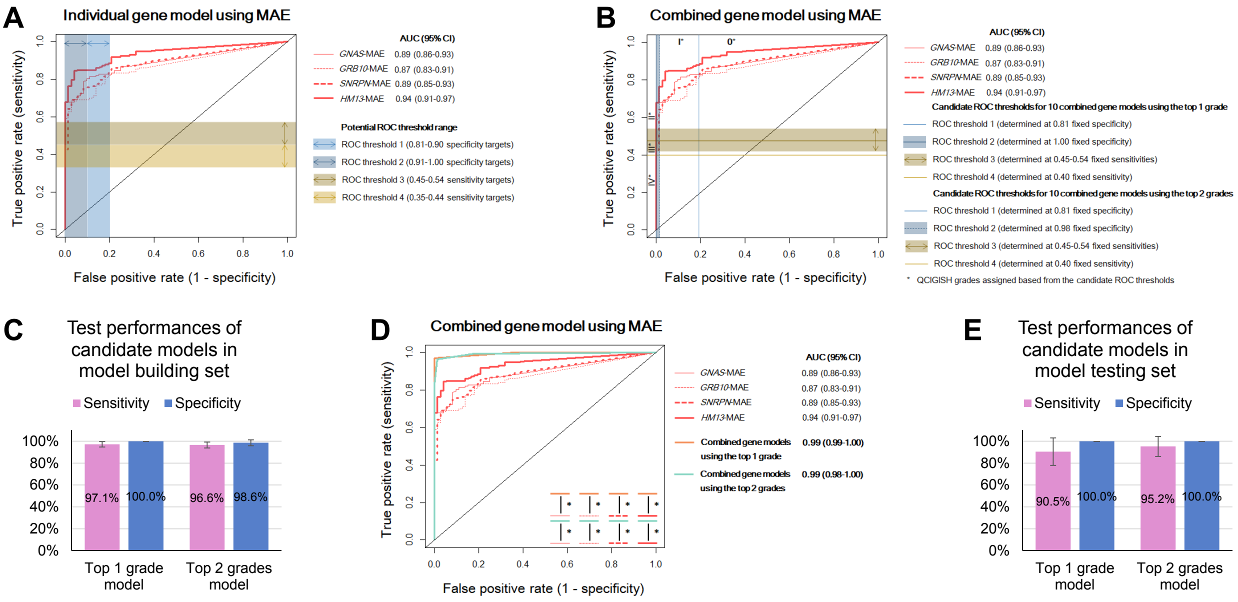
A
Individual gene model using MAE
True positive rate (sensitivity)
False positive rate (1 - specificity)
0.0
0.2
0.4
0.6
0.8
1.0
AUC (95% CI)
GNAS-MAE 0.89 (0.86-0.93)
GRB10-MAE 0.87 (0.83-0.91)
SNRPN-MAE 0.89 (0.85-0.93)
HM13-MAE 0.94 (0.91-0.97)
Potential ROC threshold range
ROC threshold 1 (0.81-0.90 specificity targets)
ROC threshold 2 (0.91-1.00 specificity targets)
ROC threshold 3 (0.45-0.54 sensitivity targets)
ROC threshold 4 (0.35-0.44 sensitivity targets)
B
Combined gene model using MAE
True positive rate (sensitivity)
False positive rate (1 - specificity)
I*
0*
II*
III*
IV*
AUC (95% CI)
GNAS-MAE 0.89 (0.86-0.93)
GRB10-MAE 0.87 (0.83-0.91)
SNRPN-MAE 0.89 (0.85-0.93)
HM13-MAE 0.94 (0.91-0.97)
Candidate ROC thresholds for 10 combined gene models using the top 1 grade
ROC threshold 1 (determined at 0.81 fixed specificity)
ROC threshold 2 (determined at 1.00 fixed specificity)
ROC threshold 3 (determined at 0.45-0.54 fixed sensitivities)
ROC threshold 4 (determined at 0.40 fixed sensitivity)
Candidate ROC thresholds for 10 combined gene models using the top 2 grades
ROC threshold 1 (determined at 0.81 fixed specificity)
ROC threshold 2 (determined at 0.98 fixed specificity)
ROC threshold 3 (determined at 0.45-0.54 fixed sensitivities)
ROC threshold 4 (determined at 0.40 fixed sensitivity)
* QCIGISH grades assigned based from the candidate ROC thresholds
C
Test performances of candidate models in model building set
Sensitivity
Specificity
100%
80%
60%
40%
20%
0%
97.1%
100.0%
96.6%
98.6%
Top 1 grade model
Top 2 grades model
D
Combined gene model using MAE
True positive rate (sensitivity)
False positive rate (1 - specificity)
AUC (95% CI)
GNAS-MAE 0.89 (0.86-0.93)
GRB10-MAE 0.87 (0.83-0.91)
SNRPN-MAE 0.89 (0.85-0.93)
HM13-MAE 0.94 (0.91-0.97)
Combined gene models using the top 1 grade 0.99 (0.99-1.00)
Combined gene models using the top 2 grades 0.99 (0.98-1.00)
E
Test performances of candidate models in model testing set
Sensitivity
Specificity
100%
80%
60%
40%
20%
0%
90.5%
100.0%
95.2%
100.0%
Top 1 grade model
Top 2 grades model

Fig. S9

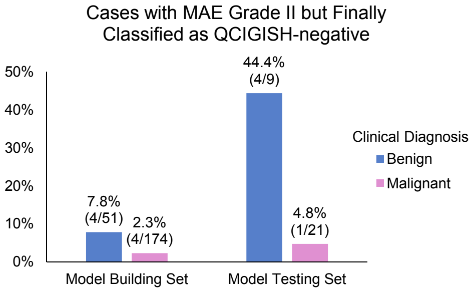
Cases with MAE Grade II but Finally
Classified as QCIGISH-negative
50%
40%
30%
20%
10%
0%
7.8%
(4/51)
2.3%
(4/174)
44.4%
(4/9)
4.8%
(1/21)
Model Building Set
Model Testing Set
Clinical Diagnosis
Benign
Malignant

Fig. S10

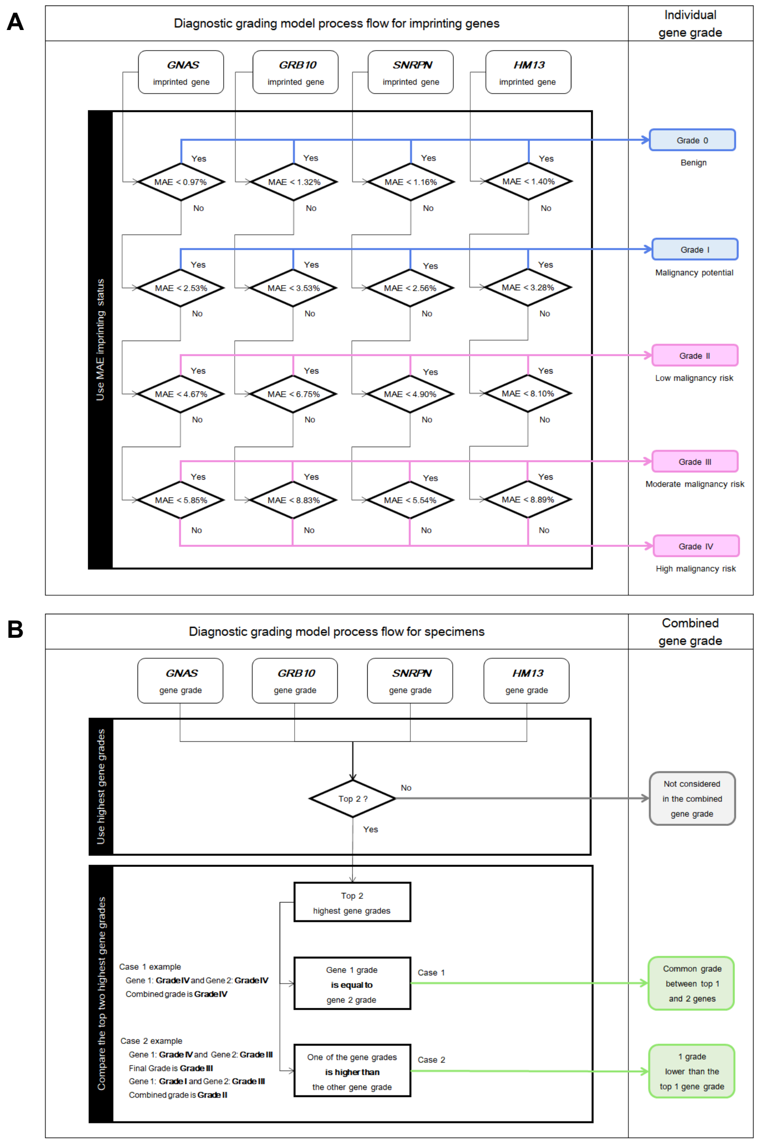
A
Diagnostic grading model process flow for imprinting genes
Individual gene grade
GNAS imprinted gene
GRB10 imprinted gene
SNRPN imprinted gene
HM13 imprinted gene
Use MAE imprinting status
MAE < 0.97%
MAE < 1.32%
MAE < 1.16%
MAE < 1.40%
MAE < 2.53%
MAE < 3.53%
MAE < 2.56%
MAE < 3.28%
MAE < 4.67%
MAE < 6.75%
MAE < 4.90%
MAE < 8.10%
MAE < 5.85%
MAE < 8.83%
MAE < 5.54%
MAE < 8.89%
Yes
No
Grade 0
Benign
Grade I
Malignancy potential
Grade II
Low malignancy risk
Grade III
Moderate malignancy risk
Grade IV
High malignancy risk
B
Diagnostic grading model process flow for specimens
Combined gene grade
GNAS gene grade
GRB10 gene grade
SNRPN gene grade
HM13 gene grade
Use highest gene grades
Top 2 ?
No
Yes
Not considered in the combined gene grade
Compare the top two highest gene grades
Top 2 highest gene grades
Case 1 example
Gene 1: Grade IV and Gene 2: Grade IV
Combined grade is Grade IV
Gene 1 grade is equal to gene 2 grade
Case 1
Common grade between top 1 and 2 genes
Case 2 example
Gene 1: Grade IV and Gene 2: Grade III
Final Grade is Grade III
Gene 1: Grade I and Gene 2: Grade III
Combined grade is Grade II
One of the gene grades is higher than the other gene grade
Case 2
1 grade lower than the top 1 gene grade

Fig. S11

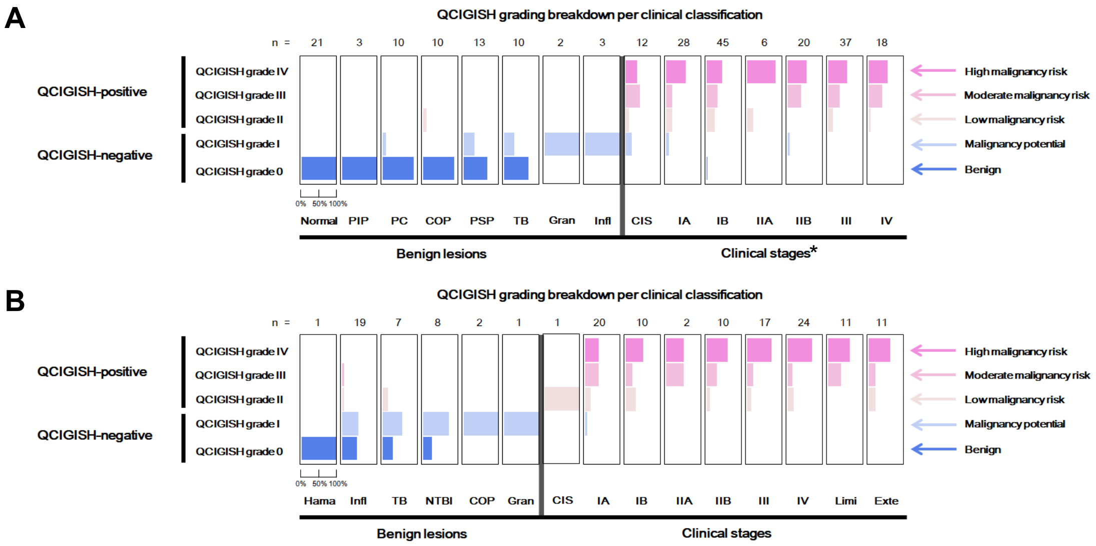
A
QCIGISH grading breakdown per clinical classification
n = 21 3 10 10 13 10 2 3 12 28 45 6 20 37 18
QCIGISH-positive
QCIGISH-negative
QCIGISH grade IV
QCIGISH grade III
QCIGISH grade II
QCIGISH grade I
QCIGISH grade 0
High malignancy risk
Moderate malignancy risk
Low malignancy risk
Malignancy potential
Benign
0% 50% 100%
Normal PIP PC COP PSP TB Gran Infl CIS IA IB IIA IIB III IV
Benign lesions
Clinical stages*
B
QCIGISH grading breakdown per clinical classification
n = 1 19 7 8 2 1 1 20 10 2 10 17 24 11 11
QCIGISH-positive
QCIGISH-negative
QCIGISH grade IV
QCIGISH grade III
QCIGISH grade II
QCIGISH grade I
QCIGISH grade 0
High malignancy risk
Moderate malignancy risk
Low malignancy risk
Malignancy potential
Benign
0% 50% 100%
Hama Infl TB NTBI COP Gran CIS IA IB IIA IIB III IV Limi Exte
Benign lesions
Clinical stages

Fig. S12

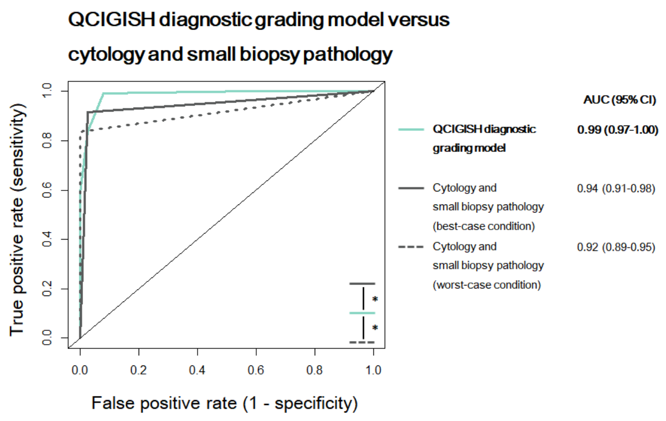
QCIGISH diagnostic grading model versus
cytology and small biopsy pathology
True positive rate (sensitivity)
False positive rate (1 - specificity)
0.0
0.2
0.4
0.6
0.8
1.0
AUC (95% CI)
QCIGISH diagnostic grading model
0.99 (0.97-1.00)
Cytology and small biopsy pathology (best-case condition)
0.94 (0.91-0.98)
Cytology and small biopsy pathology (worst-case condition)
0.92 (0.89-0.95)
*
*

Fig. S13

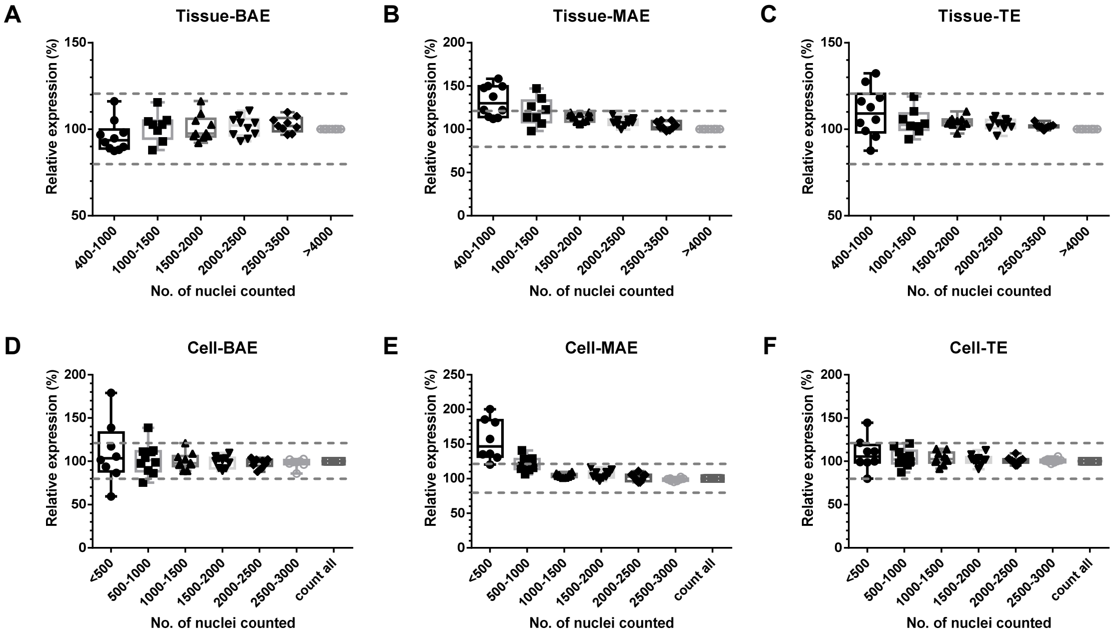

A
Tissue-BAE
B
Tissue-MAE
C
Tissue-TE
D
Cell-BAE
E
Cell-MAE
F
Cell-TE
Relative expression (%)
No. of nuclei counted
400-1000
1000-1500
1500-2000
2000-2500
2500-3500
>4000
<500
500-1000
2500-3000
count all

Fig. S14

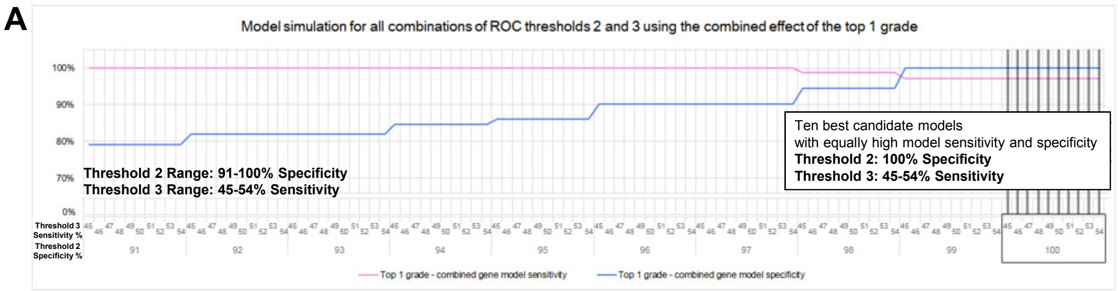
A
Model simulation for all combinations of ROC thresholds 2 and 3 using the combined effect of the top 1 grade
Ten best candidate models
with equally high model sensitivity and specificity
Threshold 2: 100% Specificity
Threshold 3: 45-54% Sensitivity
Threshold 2 Range: 91-100% Specificity
Threshold 3 Range: 45-54% Sensitivity
Threshold 3 Sensitivity %
Threshold 2 Specificity %
Top 1 grade - combined gene model sensitivity
Top 1 grade - combined gene model specificity

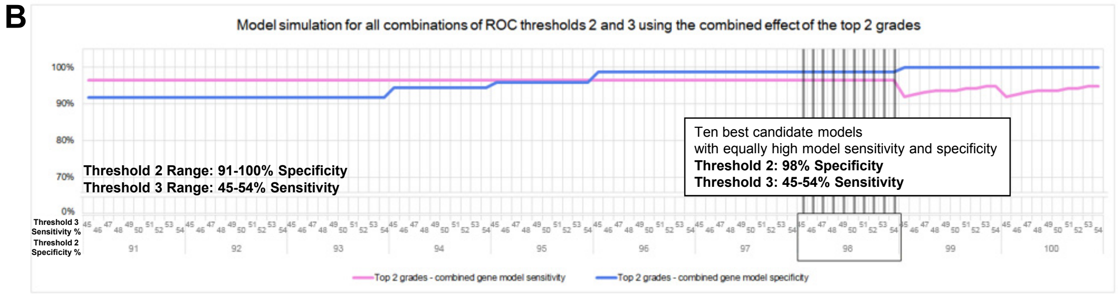
B
Model simulation for all combinations of ROC thresholds 2 and 3 using the combined effect of the top 2 grades
Ten best candidate models
with equally high model sensitivity and specificity
Threshold 2: 98% Specificity
Threshold 3: 45-54% Sensitivity
Threshold 2 Range: 91-100% Specificity
Threshold 3 Range: 45-54% Sensitivity
Threshold 3 Sensitivity %
Threshold 2 Specificity %
Top 2 grades - combined gene model sensitivity
Top 2 grades - combined gene model specificity
