# Supplementary material for: Epigenetic imprinting alterations as effective diagnostic biomarkers for early-stage lung cancer and small pulmonary nodules
Source: Clin Epigenetics. 2021 Dec 14;13:220. doi: 10.1186/s13148-021-01203-5 (PMC8672623; doi:10.1186/s13148-021-01203-5)
Supplement: Supplementary file 2 — Additional file 2. Supplementary Materials, Methods and Tables. [file 13148_2021_1203_MOESM2_ESM.docx]

**Epigenetic imprinting alterations as effective diagnostic biomarkers for early-stage lung cancer**

**Supplementary Materials:**

**Materials and Methods**

**CT Examination**

Low-dose CT examinations were performed using different multidetector scanners with a minimum of four channels. The acquisition variables were set to reduce the patient radiation exposure to an average effective dose of 1.5 mSv. Low-dose CT acquisitions were interpreted at each medical center by experienced radiologists. Patients with solid or part-solid pulmonary nodules measuring at least 8 mm in diameter or lung masses with reported diameters of at least 3 cm were proceeded for further bronchoscopy or core needle biopsy examinations[[1](#_ENREF_1)]. Patients with solid or part-solid nodules smaller than 8 mm were also recommended for bronchoscopy or core needle biopsy for cases which were evaluated as suspicious or high risk for lung cancer after a thorough clinical assessment made by experienced pulmonologists[[1](#_ENREF_1)].

**Bronchoscopy**

The patients were advised to undergo fasting for at least eight hours and received anesthesia 45 minutes prior to the clinical procedure. Bronchoscopy was then performed by experienced bronchoscopists according to a standard protocol. Small biopsy specimens were taken by pulmonary biopsy forceps. Four to six bronchial biopsies were retrieved from each patient and immediately fixed in 10% neutral buffered formalin (NBF) for 24 hours and then embedded in paraffin. The small tissue blocks were cut into 4 μm slides. The hematoxylin-eosin-stained slides were analyzed by pathologists and reported according to the WHO and IASLC/ATS/ERS guidelines[[2](#_ENREF_2), [3](#_ENREF_3)]. The bronchial brush cytology specimens were taken and analyzed according to the Papanicolaou Society of Cytopathology guidelines[[4](#_ENREF_4)].

**Transthoracic Core Needle Biopsy**

Transthoracic core needle biopsies were performed using needles ranging from 20G to 16G under CT guidance. The retrieved micro-histologic samples were immediately fixed in 10% NBF for 24 hours and then embedded in paraffin. The small tissue blocks were cut into 4 μm slides. The hematoxylin-eosin-stained slides were analyzed by pathologists and reported according to the WHO and IASLC/ATS/ERS guidelines[[2](#_ENREF_2), [3](#_ENREF_3)].

**Determination of the Minimum Nuclei Count Requirement for QCIGISH**

To determine the minimum nuclei count required for each cell or tissue sample while sufficiently maintaining the detection accuracy of allelic expressions, we randomly selected several scanned images for each sample and calculated the BAE, MAE and TE measurements using a range of experimental cell counts starting from 300 and further extending to more than 5,000 as shown in Supplementary Fig. S13. As reference, we separately evaluated the BAE, MAE and TE by considering all available cell nuclei. For each iteration, we divided the computed BAE, MAE and TE determined for the individual cell count range with the values obtained using all available cell nuclei to determine their relative expressions, respectively. From a perfect detection accuracy of 100%, we allowed the relative expressions to vary between 80% and 120% (20% measurement error). Experimental results showed high variation for low cell nuclei count (below 1000). Relative expression measurements were however stable after 1000 cell nuclei for cell samples and 1500 cells for tissue samples, which were therefore selected as the most optimal setting for QCIGISH. (Supplementary Fig. S13).

**Sample Size Computation**

The calculation for sample size was based on the assumption of 50% lung cancer prevalence among pulmonary nodules and lung masses as reported from literature[[5](#_ENREF_5)]. As a potentially new adjunctive diagnostic method, we hypothesized that QCIGISH would achieve both a sensitivity and specificity of 95% - an improvement to the 92% sensitivity and 88% specificity we have obtained from our previous study[[6](#_ENREF_6)]. For a maximum marginal error of the estimate not exceeding 5% with a 95% confidence level, the minimum required sample size was 145 patients using Buderer's formula[[7](#_ENREF_7)]. To account for a 30% dropout rate, 207 patients or more were targeted for enrollment in the blinded validation study.

**Imprinted Gene Biomarker Pre-selection**

To select the most effective biomarkers for distinguishing benign lesions from cancers, we calculated the ROC AUC for the BAE, MAE and TE measurements of the imprinted genes *GNAS*, *GRB10*, *SNRPN* and *HM13* for all 246 samples in the model building set (Supplementary Fig. S4). Optimal thresholds, using the maximized sum of sensitivity and specificity from the ROC curves, were determined and applied to dichotomize the BAE, MAE and TE numeric values into positive and negative categories (Supplementary Table S7). These categories were pooled to determine the accuracy of each marker using specificity computation for the normal and benign subtypes, and sensitivity computation for the lung cancer subtypes. To assess the effectiveness of the individual markers, computed sensitivities and specificities of at least 80% or higher were considered useful for the study.

**Diagnostic Grading Model Algorithm Development, Threshold Evaluation and Optimization**

Four thresholds on the ROC curves of MAE were defined to classify the model building set samples into five individual gene grades representative of progressive lung malignancy risks. QCIGISH diagnostic grades 0 and I are both considered as QCIGISH-negative, representing benign and malignancy potential classifications, respectively. Low, medium and high malignancy risks are all collectively classified as QCIGISH-positive and indicated by QCIGISH diagnostic grades II, III and IV, respectively. To determine the final combined grade, all four predicted grades from each gene were ranked. When only the top one grade is considered, the highest grade representing the most sensitive marker among the individual gene classifiers was used. However, when evaluating using the top two grades, we compared the two highest ranked grades: we used the highest grade in instances when both grades are equal; we applied a grade adjusted one level lower than the highest grade in cases when both grades are unequal (Supplementary Fig. S7). To determine the optimal threshold combinations, ten potential values associated with various sensitivity and specificity targets were identified for each individual threshold. Thresholds 1 and 2, with emphasis on specificity, were used to classify grades 0 and I. Thresholds 3 and 4, with emphasis on sensitivity, were used to classify grades II, III and IV. Applying a 1% increment for each threshold range, there were 10,000 possible threshold combinations evaluated independently using a combined gene grade computed from both the top one and top two highest grades from all four gene classifiers resulting to computationally simulating 20,000 possible threshold combinations. We considered the combined gene grade using only the top one and top two grades from all four gene classifiers to assess the classification accuracy for each threshold combination (Supplementary Fig. S14). From the simulation results, a defined number of candidate threshold combinations with optimal diagnostic performance were determined and were independently tested in a small group of cytology and small biopsy specimens.

**References**

1. Bai C, Choi CM, Chu CM, Anantham D, Chung-Man Ho J, Khan AZ, Lee JM, Li SY, Saenghirunvattana S, Yim A: **Evaluation of Pulmonary Nodules: Clinical Practice Consensus Guidelines for Asia**. *Chest* 2016, **150**(4):877-893.

2. Travis WD, Brambilla E, Burke AP, Marx A, Nicholson AG: **WHO Classification of Tumours of the Lung, Pleura, Thymus and Heart, 4th edition**. Lyon, France: IARC Press; 2015.

3. Travis WD, Brambilla E, Noguchi M, Nicholson AG, Geisinger K, Yatabe Y, Ishikawa Y, Wistuba I, Flieder DB, Franklin W *et al*: **Diagnosis of lung cancer in small biopsies and cytology: implications of the 2011 International Association for the Study of Lung Cancer/American Thoracic Society/European Respiratory Society classification**. *Archives of pathology & laboratory medicine* 2013, **137**(5):668-684.

4. Layfield LJ, Baloch Z, Elsheikh T, Litzky L, Rekhtman N, Travis WD, Zakowski M, Zarka M, Geisinger K: **Standardized terminology and nomenclature for respiratory cytology: The Papanicolaou Society of Cytopathology guidelines**. *Diagnostic cytopathology* 2016, **44**(5):399-409.

5. Wahidi MM, Govert JA, Goudar RK, Gould MK, McCrory DC: **Evidence for the treatment of patients with pulmonary nodules: when is it lung cancer?: ACCP evidence-based clinical practice guidelines (2nd edition)**. *Chest* 2007, **132**(3 Suppl):94s-107s.

6. Shen R, Cheng T, Xu C, Yung RC, Bao J, Li X, Yu H, Lu S, Xu H, Wu H *et al*: **Novel visualized quantitative epigenetic imprinted gene biomarkers diagnose the malignancy of ten cancer types**. *Clinical epigenetics* 2020, **12**(1):71.

7. Buderer NM: **Statistical methodology: I. Incorporating the prevalence of disease into the sample size calculation for sensitivity and specificity**. *Academic emergency medicine : official journal of the Society for Academic Emergency Medicine* 1996, **3**(9):895-900.

**Supplement Figure Legends**

**Fig. S1. STARD diagram showing the subset and flow of participants through the study.** *, a special subset of 60 cases was randomly sampled from the model building set for a preliminary gene selection study.

**Fig. S2. Timeline chart showing the coverage dates of the enrolled samples for the model building, testing and blinded validation sets.**

**Fig. S3. Differential analysis of imprinted gene expression in normal, benign and malignant lung tissue specimens. A,** Heatmap analysis on the imprinted gene panel showing elevated relative allelic expression patterns involving BAE, MAE and TE for the lung cancers as compared to the benign lesions and normal controls. **B,** Statistical evaluation showing significant differences in the BAE, MAE and TE status for the imprinted gene panel between the normal, benign, and malignant cases from the model building set. *, significant differences between groups, Bonferroni-adjusted *p* < 0.05. n.s., not significant.

**Fig. S4. ROC curves showing improved malignancy discrimination using MAE for imprinted genes *GNAS*, *GRB10*, *SNRPN* and *HM13* in the model building set.**

**Fig. S5. Differential sensitivity and specificity analysis of imprinted genes *GNAS*, *GRB10*, *SNRPN* and *HM13* pooled for different subtypes of benign and malignant lung lesions in the model building set.**

**Fig. S6. Modeling framework.**

**Fig. S7. Grading model algorithm.**

**Fig. S8. QCIGISH model threshold evaluation and accuracy estimation. A,** ROC curves showing the ranges for selecting model thresholds 1, 2, 3 and 4 for the MAE of each imprinted gene in the model building set. **B,** ROC curves showing fixed model thresholds 1 and 4 and the ranges for selecting model thresholds 2 and 3 for the MAE of each imprinted gene. **C,** Optimal sensitivities and specificities derived from the top 1 grade model and top 2 grade model in the model building set. **D,** ROC curves showing improved malignancy discrimination for the candidate models using the top 1 or top 2 imprinted genes as compared to the individual MAE measurements for all imprinted genes in the model building set. *, significant differences between AUC values, *p* < 0.05. **E,** Optimal sensitivities and specificities derived from the top 1 grade model and top 2 grade model in the model testing set. Error bars represent the 95% confidence interval for the computed sensitivities and specificities.

**Fig. S9. Cases with highest grade reported as grade II but adjusted to grade I in the model building and testing set.**

**Fig. S10. Diagnostic grading model.** **A,** Diagnostic grading flow for genes. **B,** Diagnostic grading flow for samples.

**Fig. S11. QCIGISH grade distribution in different benign lung lesion sybtypes and lung cancer stages.** **A,** QCIGISH diagnostic grade breakdown in different benign lung lesion subtypes and lung cancer stages in the model building set. *8 NSCLC cases (7 SqCC, 1 LCC) with insufficient clinical staging information were excluded from the plot. **B,** QCIGISH diagnostic grade breakdown in different benign lung lesion subtypes and lung cancer stages in the model validation set.

**Fig. S12. ROC curves showing improved malignancy discrimination for the QCIGISH diagnostic grading model as compared to the cytology and small biopsy pathology diagnosis in the model validation set.** Best-case conditions indicate that indeterminate cytology and small biopsy cases were classified as positive during comparison. Worst-case conditions indicate that indeterminate cytology and small biopsy cases were classified as negative during comparison. *, significant differences between AUC values, *p* < 0.05.

**Fig. S13. Sensitivity analysis of BAE, MAE and TE measurement variations across a range of cell nuclei counts. A-C,** BAE (A), MAE (B) and TE (C) measurement variations in tissue samples. **D-F,** BAE (D), MAE (E) and TE (F) measurement variations in cell samples. The 20% measurement error interval was indicated by two dashed lines representing 80% and 120%, respectively.

**Fig. S14. Computational simulation results using variable model thresholds 2 and 3 in the model building set.** **A,** Best candidate models using the top 1 grade determined for 100% specificity and 45-54% sensitivity targets. **B,** Best candidate models using the top 2 grades determined for 98% specificity and 45-54% sensitivity targets.

**Supplement Tables**

**Table S1.** Baseline clinical characteristics of the gene selection set.

|  | **Gene Selection Set**  **(*n* = 60)** | | | **Model Building Set**  **(*n* = 246)** | | | |
| --- | --- | --- | --- | --- | --- | --- | --- |
|  | **Benign** | **Malignant** | ***p*** | **Normal^A^** | **Benign** | **Malignant** | ***p*** |
|  | **(*n* = 30)** | **(*n* = 30)** |  | **(*n* = 21)** | **(*n* = 51)** | **(*n* = 174)** |  |
| **Age** |  |  | 0.034 |  |  |  | <0.001 |
| Median | 57 | 64 |  | 52 | 57 | 62 |  |
| IQR | 51 to 63 | 54 to 66 |  | 45 to 57 | 49 to 63 | 53 to 67 |  |
| **Sex (%)** |  |  | 0.429 |  |  |  | 0.467 |
| Male | 14 (46.7%) | 10 (33.3%) |  | 14 (66.7%) | 28 (54.9%) | 111 (63.8%) |  |
| Female | 16 (53.3%) | 20 (66.7%) |  | 7 (33.3%) | 23 (45.1%) | 63 (36.2%) |  |
| **Sample type** |  |  |  |  |  |  |  |
| Surgically resected  tissue specimen | 30 | 30 |  | 21 | 51 | 174 |  |
| Small biopsy specimen |  |  |  |  |  |  |  |
| Cytology specimen |  |  |  |  |  |  |  |
| **Histologic characteristics no.** |  |  |  |  |  |  |  |
| Normal |  |  |  | 21 |  |  |  |
| PC | 3 |  |  |  | 10 |  |  |
| PSP | 8 |  |  |  | 13 |  |  |
| TB | 8 |  |  |  | 10 |  |  |
| COP | 5 |  |  |  | 10 |  |  |
| PIP | 2 |  |  |  | 3 |  |  |
| Non-TB infections |  |  |  |  |  |  |  |
| Inflammation | 2 |  |  |  | 3 |  |  |
| Granuloma | 2 |  |  |  | 2 |  |  |
| Hamartoma |  |  |  |  |  |  |  |
| AdC |  | 15 |  |  |  | 94 |  |
| SqCC |  | 15 |  |  |  | 76 |  |
| AdSqLC |  |  |  |  |  | 2 |  |
| LCC |  |  |  |  |  | 1 |  |
| SCLC |  |  |  |  |  |  |  |
| Carcinoma of  unknown primary |  |  |  |  |  | 1 |  |
| **Nodule size** |  |  | ^E^ |  |  |  | ^E^ |
| <0.8 cm |  |  |  |  |  | 7 |  |
| ≥0.8-2.0 cm | 1 | 8 |  |  | 3 | 35 |  |
| >2.0-3.0 cm | 0 | 8 |  |  |  | 48 |  |
| >3.0-5.0 cm | 1 | 11 |  |  | 1 | 52 |  |
| >5.0 cm | 0 | 3 |  |  |  | 28 |  |
| Unclear LDCT  features^B^ | 26 | 0 |  | 21 | 44 |  |  |
| Not specified^C^ | 2 | 0 |  |  | 3 | 4 |  |
| **Smoking status^D^ (%)** |  |  | 0.039 |  |  |  | 0.001 |
| Current smoker | 2 (6.7%) | 8 (26.7%) |  | 3 (14.3%) | 3 (5.9%) | 56 (32.2%) |  |
| Former smoker | 0 (0.0%) | 2 (6.7%) |  | 1 (4.8%) | 1 (2.0%) | 11 (6.3%) |  |
| Non-smoker | 23 (76.7%) | 17 (56.7%) |  | 15 (71.4%) | 40 (78.4%) | 90 (51.7%) |  |
| Not specified | 5 (16.7%) | 3 (10.0%) |  | 2 (9.5%) | 7 (13.7%) | 17 (9.8%) |  |

^A^, normal tissue specimens were resected adjacent to the benign lesions. ^B^, no typical nodule under LDCT. ^C^, nodule sizes not recorded by doctors. ^D^, cases classified as current or former smokers were combined into a single category prior to analysis to comply with the statistical test requirements. ^E^, no analysis proceeded since data transformations applied failed to meet the statistical test requirements. PC, pulmonary cryptococcosis. PSP, pulmonary sclerosing pneumocytoma. TB, pulmonary tuberculosis. COP, cryptogenic organizing pneumonia. PIP, pulmonary inflammatory pseudotumor. AdC, adenocarcinoma. SqCC, squamous cell carcinoma. AdSqLC, adenosquamous lung carcinoma. LCC, large cell carcinoma. SCLC, small cell lung cancer.

**Table S2. ROC comparison of the diagnostic performance of the QCIGISH binary classification model^A^ and individual imprinted gene *HM13* BAE, MAE and TE measurements**

| **Diagnostic Performance Comparative Analysis** | | **Computed *p*** |
| --- | --- | --- |
| **Method** | **ROC AUC (95% CI)** |  |
| QCIGISH binary classification model | 0.88333 (0.80080–0.96580) | **0.04368*** |
| *HM13*-BAE | 0.94667 (0.88810-1.00000) |  |
| QCIGISH binary classification model | 0.88333 (0.80080–0.96580) | **0.00782*** |
| *HM13*-MAE | 0.98222 (0.95230-1.00000) |  |
| QCIGISH binary classification model | 0.88333 (0.80080–0.96580) | **0.51140** |
| *HM13*-TE | 0.85556 (0.76150-0.949600) |  |

* Computed *p* significant at alpha = 0.05

^A^ Malignancy prediction using the combined BAE, MAE and TE measurements of the *GNAS*, *GRB10* and *SNRPN* imprinted gene panel

**Table S3. Comparison of BAE, MAE and TE status between normal, benign and malignant cases for *GNAS*.**

| ***GNAS*-BAE statistical evaluation by independent groups** | | | | **Computed *p*** |
| --- | --- | --- | --- | --- |
| **Lung lesion**  **clinical classification** | **n** | **Median** | **IQR** |  |
| Normal | 21 | 0.00 | 0.00 | **0.00000***  **(<2.20e-16)** |
| Benign | 51 | 5.05 | 6.11 |  |
| Malignant | 174 | 17.50 | 13.10 |  |
| Normal | 21 | 0.00 | 0.00 | **0.03392**** |
| Benign | 51 | 5.05 | 6.11 |  |
| Normal | 21 | 0.00 | 0.00 | **0.00000****  **(1.36e-14)** |
| Malignant | 174 | 17.50 | 13.10 |  |
| Benign | 51 | 5.05 | 6.11 | **0.00000****  **(1.26e-12)** |
| Malignant | 174 | 17.50 | 13.10 |  |
| ***GNAS*-MAE statistical evaluation by independent groups** | | | | **Computed *p*** |
| **Lung lesion**  **clinical classification** | **n** | **Median** | **IQR** |  |
| Normal | 21 | 0.00 | 0.00 | **0.00000***  **(<2.20e-16)** |
| Benign | 51 | 0.00 | 0.98 |  |
| Malignant | 174 | 4.13 | 7.62 |  |
| Normal | 21 | 0.00 | 0.00 | **0.56086** |
| Benign | 51 | 0.00 | 0.98 |  |
| Normal | 21 | 0.00 | 0.00 | **0.00000****  **(6.38e-12)** |
| Malignant | 174 | 4.13 | 7.62 |  |
| Benign | 51 | 0.00 | 0.98 | **0.00000****  **(2.61e-15)** |
| Malignant | 174 | 4.13 | 7.62 |  |
| ***GNAS*-TE statistical evaluation by independent groups** | | | | **Computed *p*** |
| **Lung lesion**  **clinical classification** | **n** | **Median** | **IQR** |  |
| Normal | 21 | 1.35 | 2.74 | **0.00000***  **(<2.20e-16)** |
| Benign | 51 | 6.69 | 19.40 |  |
| Malignant | 174 | 29.80 | 30.00 |  |
| Normal | 21 | 1.35 | 2.74 | **0.00542**** |
| Benign | 51 | 6.69 | 19.40 |  |
| Normal | 21 | 1.35 | 2.74 | **0.00000****  **(6.64e-14)** |
| Malignant | 174 | 29.80 | 30.00 |  |
| Benign | 51 | 6.69 | 19.40 | **0.00000****  **(5.94e-09)** |
| Malignant | 174 | 29.80 | 30.00 |  |

* Significant difference (*p* < 0.05) between the Normal, Benign and Malignant groups

** Significant difference (Bonferroni-adjusted *p* < 0.05) between paired groups

**Table S4. Comparison of BAE, MAE and TE status between normal, benign and malignant cases for *GRB10*.**

| ***GRB10*-BAE statistical evaluation by independent groups** | | | | **Computed *p*** |
| --- | --- | --- | --- | --- |
| **Lung lesion**  **clinical classification** | **n** | **Median** | **IQR** |  |
| Normal | 21 | 0.00 | 0.00 | **0.00000***  **(<2.20e-16)** |
| Benign | 51 | 7.69 | 8.90 |  |
| Malignant | 174 | 17.30 | 10.90 |  |
| Normal | 21 | 0.00 | 0.00 | **0.00272**** |
| Benign | 51 | 7.69 | 8.90 |  |
| Normal | 21 | 0.00 | 0.00 | **0.00000****  **(1.68e-14)** |
| Malignant | 174 | 17.30 | 10.90 |  |
| Benign | 51 | 7.69 | 8.90 | **0.00000****  **(8.98e-09)** |
| Malignant | 174 | 17.30 | 10.90 |  |
| ***GRB10*-MAE statistical evaluation by independent groups** | | | | **Computed *p*** |
| **Lung lesion**  **clinical classification** | **n** | **Median** | **IQR** |  |
| Normal | 21 | 0.00 | 0.00 | **0.00000***  **(<2.20e-16)** |
| Benign | 51 | 0.16 | 1.47 |  |
| Malignant | 174 | 6.26 | 9.69 |  |
| Normal | 21 | 0.00 | 0.00 | **0.21563** |
| Benign | 51 | 0.16 | 1.47 |  |
| Normal | 21 | 0.00 | 0.00 | **0.00000****  **(4.81e-12)** |
| Malignant | 174 | 6.26 | 9.69 |  |
| Benign | 51 | 0.16 | 1.47 | **0.00000****  **(7.49e-13)** |
| Malignant | 174 | 6.26 | 9.69 |  |
| ***GRB10*-TE statistical evaluation by independent groups** | | | | **Computed *p*** |
| **Lung lesion**  **clinical classification** | **n** | **Median** | **IQR** |  |
| Normal | 21 | 2.25 | 2.04 | **0.00000***  **(6.84e-14)** |
| Benign | 51 | 9.03 | 19.10 |  |
| Malignant | 174 | 24.20 | 25.80 |  |
| Normal | 21 | 2.25 | 2.04 | **0.00101**** |
| Benign | 51 | 9.03 | 19.10 |  |
| Normal | 21 | 2.25 | 2.04 | **0.00000****  **(5.66e-12)** |
| Malignant | 174 | 24.20 | 25.80 |  |
| Benign | 51 | 9.03 | 19.10 | **0.00003**** |
| Malignant | 174 | 24.20 | 25.80 |  |

* Significant difference (*p* < 0.05) between the Normal, Benign and Malignant groups

** Significant difference (Bonferroni-adjusted *p* < 0.05) between paired groups

**Table S5. Comparison of BAE, MAE and TE status between normal, benign and malignant cases for *SNRPN*.**

| ***SNRPN*-BAE statistical evaluation by independent groups** | | | | **Computed *p*** |
| --- | --- | --- | --- | --- |
| **Lung lesion**  **clinical classification** | **n** | **Median** | **IQR** |  |
| Normal | 21 | 0.00 | 0.00 | **0.00000***  **(<2.20e-16)** |
| Benign | 51 | 6.09 | 7.66 |  |
| Malignant | 174 | 17.50 | 11.10 |  |
| Normal | 21 | 0.00 | 0.00 | **0.01007**** |
| Benign | 51 | 6.09 | 7.66 |  |
| Normal | 21 | 0.00 | 0.00 | **0.00000****  **(6.07e-15)** |
| Malignant | 174 | 17.50 | 11.10 |  |
| Benign | 51 | 6.09 | 7.66 | **0.00000****  **(4.65e-11)** |
| Malignant | 174 | 17.50 | 11.10 |  |
| ***SNRPN*-MAE statistical evaluation by independent groups** | | | | **Computed *p*** |
| **Lung lesion**  **clinical classification** | **n** | **Median** | **IQR** |  |
| Normal | 21 | 0.00 | 0.00 | **0.00000***  **(<2.20e-16)** |
| Benign | 51 | 0.29 | 1.17 |  |
| Malignant | 174 | 4.31 | 6.23 |  |
| Normal | 21 | 0.00 | 0.00 | **0.23782** |
| Benign | 51 | 0.29 | 1.17 |  |
| Normal | 21 | 0.00 | 0.00 | **0.00000****  **(1.01e-12)** |
| Malignant | 174 | 4.31 | 6.23 |  |
| Benign | 51 | 0.29 | 1.17 | **0.00000****  **(4.00e-14)** |
| Malignant | 174 | 4.31 | 6.23 |  |
| ***SNRPN*-TE statistical evaluation by independent groups** | | | | **Computed *p*** |
| **Lung lesion**  **clinical classification** | **n** | **Median** | **IQR** |  |
| Normal | 21 | 1.13 | 2.54 | **0.00000***  **(2.66e-16)** |
| Benign | 51 | 16.10 | 25.40 |  |
| Malignant | 174 | 31.70 | 26.50 |  |
| Normal | 21 | 1.13 | 2.54 | **0.00012**** |
| Benign | 51 | 16.10 | 25.40 |  |
| Normal | 21 | 1.13 | 2.54 | **0.00000****  **(2.52e-14)** |
| Malignant | 174 | 31.70 | 26.50 |  |
| Benign | 51 | 16.10 | 25.40 | **0.00002**** |
| Malignant | 174 | 31.70 | 26.50 |  |

* Significant difference (*p* < 0.05) between the Normal, Benign and Malignant groups

** Significant difference (Bonferroni-adjusted *p* < 0.05) between paired groups

**Table S6. Comparison of BAE, MAE and TE status between normal, benign and malignant cases for *HM13*.**

| ***HM13*-BAE statistical evaluation by independent groups** | | | | **Computed *p*** |
| --- | --- | --- | --- | --- |
| **Lung lesion**  **clinical classification** | **n** | **Median** | **IQR** |  |
| Normal | 21 | 0.00 | 0.00 | **0.00000***  **(<2.20e-16)** |
| Benign | 51 | 6.89 | 8.70 |  |
| Malignant | 174 | 23.30 | 9.02 |  |
| Normal | 21 | 0.00 | 0.00 | **0.04122**** |
| Benign | 51 | 6.89 | 8.70 |  |
| Normal | 21 | 0.00 | 0.00 | **0.00000****  **(1.27e-16)** |
| Malignant | 174 | 23.30 | 9.02 |  |
| Benign | 51 | 6.89 | 8.70 | **0.00000****  **(8.30e-16)** |
| Malignant | 174 | 23.30 | 9.02 |  |
| ***HM13*-MAE statistical evaluation by independent groups** | | | | **Computed *p*** |
| **Lung lesion**  **clinical classification** | **n** | **Median** | **IQR** |  |
| Normal | 21 | 0.00 | 0.00 | **0.00000***  **(<2.20e-16)** |
| Benign | 51 | 0.29 | 1.46 |  |
| Malignant | 174 | 7.62 | 9.61 |  |
| Normal | 21 | 0.00 | 0.00 | **0.34835** |
| Benign | 51 | 0.29 | 1.46 |  |
| Normal | 21 | 0.00 | 0.00 | **0.00000****  **(1.10e-14)** |
| Malignant | 174 | 7.62 | 9.61 |  |
| Benign | 51 | 0.29 | 1.46 | **0.00000****  **(2.57e-18)** |
| Malignant | 174 | 7.62 | 9.61 |  |
| ***HM13*-TE statistical evaluation by independent groups** | | | | **Computed *p*** |
| **Lung lesion**  **clinical classification** | **n** | **Median** | **IQR** |  |
| Normal | 21 | 1.34 | 3.03 | **0.00000***  **(<2.20e-16)** |
| Benign | 51 | 19.40 | 28.10 |  |
| Malignant | 174 | 47.50 | 27.60 |  |
| Normal | 21 | 1.34 | 3.03 | **0.00142**** |
| Benign | 51 | 19.40 | 28.10 |  |
| Normal | 21 | 1.34 | 3.03 | **0.00000****  **(8.67e-16)** |
| Malignant | 174 | 47.50 | 27.60 |  |
| Benign | 51 | 19.40 | 28.10 | **0.00000****  **(2.01e-09)** |
| Malignant | 174 | 47.50 | 27.60 |  |

* Significant difference (*p* < 0.05) between the Normal, Benign and Malignant groups

** Significant difference (Bonferroni-adjusted *p* < 0.05) between paired groups

**Table S7. Model building set lung lesion BAE, MAE and TE optimal thresholds using maximum sensitivity and specificity from the receiver operating characteristics curve for the individual effects of imprinted genes.**

| **ROC thresholds for lung malignancy differentiation using optimal sensitivity and specificity** | | | | |
| --- | --- | --- | --- | --- |
| **Imprinted gene** | **Imprinting status** | **ROC threshold** | **Maximum (sensitivity + specificity)** | |
|  |  |  | **Sensitivity** | **Specificity** |
| ***GNAS*** | BAE | 10.35% | 0.77586 | 0.90278 |
|  | MAE | 1.35% | 0.80460 | 0.90278 |
|  | TE | 12.13% | 0.85057 | 0.76389 |
| ***GRB10*** | BAE | 13.31% | 0.68391 | 0.87500 |
|  | MAE | 2.85% | 0.68391 | 0.97222 |
|  | TE | 9.69% | 0.81609 | 0.69444 |
| ***SNRPN*** | BAE | 8.54% | 0.86207 | 0.76389 |
|  | MAE | 1.71% | 0.75862 | 0.91667 |
|  | TE | 7.85% | 0.93678 | 0.56944 |
| ***HM13*** | BAE | 13.67% | 0.89080 | 0.84722 |
|  | MAE | 2.10% | 0.84483 | 0.95833 |
|  | TE | 24.20% | 0.88506 | 0.72222 |

**Table S8. Computational simulation of grading model thresholds in the model building set.**

|  | | **Combined gene model** | |
| --- | --- | --- | --- |
| **Optimal ROC thresholds based from simulation study** | | **Using the top 1 grade** | **Using the top 2 grades** |
| ROC threshold 1 | | 0.81 to 0.90 | 0.81 to 0.90 |
| ROC threshold 2 | | 0.99 to 1.00 | 0.96 to 0.98 |
| ROC threshold 3 | | 0.45 to 0.54 | 0.45 to 0.54 |
| ROC threshold 4 | | 0.35 to 0.44 | 0.35 to 0.44 |
| **Optimal accuracy** | **Sensitivity (95% CI)** | **97.13%**  **(94.64%-99.61%)** | **96.55%**  **(93.84%-99.26%)** |
|  | **Specificity (95% CI)** | **100.00%** | **98.61%**  **(95.91%-100.00%)** |
| **Total number of potential models with optimal accuracy** | | **2000 / 10000** | **3000 / 10000** |
| **Number of selected candidate models with optimal accuracy** | | **10* / 2000** | **10* / 3000** |

*ROC thresholds 1 and 4 were fixed with only ROC thresholds 2 and 3 allowed to vary.

**Table S9. ROC comparison of the diagnostic performance of the QCIGISH diagnostic grading model using the Top 1 or 2 combined imprinted gene grades and individual imprinted gene MAE measurements**

| **Diagnostic Performance Comparative Analysis** | | **Computed *p*** |
| --- | --- | --- |
| **Method** | **ROC AUC (95% CI)** |  |
| Combined gene model using the Top 1 grade | 0.99481 (0.99000–0.99960) | **0.00000***  **(1.05e-08)** |
| *GNAS*-MAE | 0.89368 (0.85740-0.92990) |  |
| Combined gene model using the Top 1 grade | 0.99481 (0.99000–0.99960) | **0.00000***  **(1.12e-09)** |
| *GRB10*-MAE | 0.87213 (0.83140-0.91280) |  |
| Combined gene model using the Top 1 grade | 0.99481 (0.99000–0.99960) | **0.00000***  **(8.60e-08)** |
| *SNRPN*-MAE | 0.88953 (0.85090-0.92820) |  |
| Combined gene model using the Top 1 grade | 0.99481 (0.99000–0.99960) | **0.00013*** |
| *HM13*-MAE | 0.94001 (0.91270-0.96730) |  |
| Combined gene model using the Top 2 grades | 0.99286 (0.98520–1.0000) | **0.00000***  **(2.32e-08)** |
| *GNAS*-MAE | 0.89368 (0.85740-0.92990) |  |
| Combined gene model using the Top 2 grades | 0.99286 (0.98520–1.0000) | **0.00000***  **(3.79e-09)** |
| *GRB10*-MAE | 0.87213 (0.83140-0.91280) |  |
| Combined gene model using the Top 2 grades | 0.99286 (0.98520–1.0000) | **0.00000***  **(7.43e-08)** |
| *SNRPN*-MAE | 0.88953 (0.85090-0.92820) |  |
| Combined gene model using the Top 2 grades | 0.99286 (0.98520–1.0000) | **0.00012*** |
| *HM13*-MAE | 0.94001 (0.91270-0.96730) |  |

* Computed *p* significant at alpha = 0.05

**Table S10. Computational simulation of grading model thresholds in the model testing set.**

|  | | **Combined gene model** | |
| --- | --- | --- | --- |
| **Optimal ROC thresholds based from simulation study** | | **Using the top 1 grade** | **Using the top 2 grades** |
| ROC threshold 1 | | 0.81 | 0.81 |
| ROC threshold 2 | | 1.00 | 0.98 |
| ROC threshold 3 | | 0.45 to 0.54 | 0.45 to 0.54 |
| ROC threshold 4 | | 0.40 | 0.40 |
| **Optimal accuracy** | **Sensitivity (95% CI)** | **90.48%**  **(77.92%-100.00%)** | **95.24%**  **(86.13%-100.00%)** |
|  | **Specificity (95% CI)** | **100.00%** | **100.00%** |

**Table S11. Model building set lung lesion MAE diagnostic grading thresholds using optimal sensitivity and specificity targets from the receiver operating characteristics curve for the combined effects of imprinted genes.**

| **ROC thresholds for lung malignancy differentiation using fixed sensitivity or specificity targets** | | | | |
| --- | --- | --- | --- | --- |
| **Imprinted gene – imprinting status** | **Combined**  **gene model**  **threshold** | **ROC threshold** | **Individual**  **gene model sensitivity target** | **Individual**  **gene model specificity target** |
| ***GNAS* - MAE** | Threshold 1 | 0.97% | - | 0.81000* |
|  | Threshold 2 | 2.53% | - | 0.98000* |
|  | Threshold 3 | 4.67% | 0.46000* | - |
|  | Threshold 4 | 5.85% | 0.40000* | - |
| ***GRB10 -* MAE** | Threshold 1 | 1.32% | - | 0.81000* |
|  | Threshold 2 | 3.53% | - | 0.98000* |
|  | Threshold 3 | 6.75% | 0.46000* | - |
|  | Threshold 4 | 8.83% | 0.40000* | - |
| ***SNRPN-*MAE** | Threshold 1 | 1.16% | - | 0.81000* |
|  | Threshold 2 | 2.56% | - | 0.98000* |
|  | Threshold 3 | 4.90% | 0.46000* | - |
|  | Threshold 4 | 5.54% | 0.40000* | - |
| ***HM13-*MAE** | Threshold 1 | 1.40% | - | 0.81000* |
|  | Threshold 2 | 3.28% | - | 0.98000* |
|  | Threshold 3 | 8.10% | 0.46000* | - |
|  | Threshold 4 | 8.89% | 0.40000* | - |

* Fixed specificity or sensitivity targets used to determine the diagnostic grading thresholds

**Table S12. Comparison of diagnostic sensitivities and specificities of QCIGISH and cytology and small biopsy pathology.**

| **Postoperative histopathology / Clinical identification** | **QCIGISH** | | | **Cytology and small biopsy pathology** | | | | **BCC^D^**  ***p*** | **WCC^E^**  ***p*** |
| --- | --- | --- | --- | --- | --- | --- | --- | --- | --- |
|  | **Positive** | **Negative** | **Specificity^A^**  **Sensitivity^B^**  **% (95% CI)** | **Positive** | **Indeterminate^C^** | **Negative** | **Specificity^A^**  **Sensitivity^B^**  **% (95% CI)** |  |  |
| TB | 1 | 6 | 85.7 (60.0-100.0) | 0 | 0 | 7 | 100.0 | 1.000 | 1.000 |
| COP | 0 | 2 | 100.0 | 0 | 0 | 2 | 100.0 | ^F^ | ^F^ |
| Non-TB infections | 0 | 8 | 100.0 | 0 | 0 | 8 | 100.0 | ^F^ | ^F^ |
| Inflammation | 2 | 17 | 89.5 (75.7-100.0) | 0 | 1 | 18 | 94.7 (84.7-100.0) | 1.000 | 0.480 |
| Granuloma | 0 | 1 | 100.0 | 0 | 0 | 1 | 100.0 | ^F^ | ^F^ |
| Hamartoma | 0 | 1 | 100.0 | 0 | 0 | 1 | 100.0 | ^F^ | ^F^ |
| **All benign lesions^A^** | 3 | 35 | 92.1 (83.5-100.0) | 0 | 1 | 37 | 97.4 (92.3-100.0) | 0.480 | 0.248 |
| Cancer type |  |  |  |  |  |  |  |  |  |
| Adenocarcinoma | 60 | 1 | 98.4 (95.2-100.0) | 48 | 6 | 7 | 78.7 (68.4-89.0) | 0.041 | 0.001 |
| Squamous cell carcinoma | 28 | 0 | 100.0 | 23 | 3 | 2 | 82.1 (68.0-96.3) | 0.480 | 0.074 |
| Adenosquamous carcinoma | 2 | 0 | 100.0 | 2 | 0 | 0 | 100.0 | ^F^ | ^F^ |
| Small cell carcinoma | 23 | 0 | 100.0 | 22 | 0 | 1 | 95.7 (87.3-100.0) | 1.000 | 1.000 |
| Carcinoma of unknown primary | 3 | 0 | 100.0 | 2 | 0 | 1 | 66.7 (13.3-100.0) | 1.000 | 1.000 |
| **All cancers^B^** | 116 | 1 | 99.1 (97.5-100.0) | 97 | 9 | 11 | 82.9 (76.1-89.7) | 0.004 | <0.001 |

^A^, diagnostic specificity evaluated for benign lesions. ^B^, diagnostic sensitivity evaluated for lung cancers. ^C^, considered as incorrect classification during diagnostic sensitivity and specificity computation. ^D^, BCC refers to the best-case condition where the indeterminate cytology and small biopsy cases were classified as positive during diagnostic accuracy comparison with QCIGISH. ^E^, WCC refers to the worst-case condition where the indeterminate cytology and small biopsy cases were classified as negative during diagnostic accuracy comparison with QCIGISH. ^F^, complete agreement between the results of both diagnostic tests.

**Table S13. Sensitivity and specificity of QCIGISH detection in different clinical characteristics.**

|  | **Malignant** | | **Benign** | | **QCIGISH detection performance** | |
| --- | --- | --- | --- | --- | --- | --- |
| **Clinical Characteristics** | **QCIGISH-positive** | **QCIGISH-negative** | **QCIGISH-positive** | **QCIGISH-negative** | **Sensitivity,**  **% (95% CI)** | **Specificity,**  **% (95% CI)** |
| **Smoking history** | | | | | | |
| **Current smoker** | 48 | 0 | 0 | 7 | 100.0 | 100.0 |
| **Former smoker** | 11 | 0 | 0 | 2 | 100.0 | 100.0 |
| **Non-smoker** | 40 | 0 | 3 | 13 | 100.0 | 81.3 (62.1-100.0) |
| **Not specified** | 17 | 1 | 0 | 13 | 94.4 (83.9-100.0) | 100.0 |
| **Sex** | | | | | | |
| **Male** | 81 | 0 | 2 | 24 | 100.0 | 92.3 (82.1-100.0) |
| **Female** | 35 | 1 | 1 | 11 | 97.2 (91.9-100.0) | 91.7 (76.0-100.0) |
| **Age** | | | | | | |
| **31-40** | 2 | 0 | 0 | 1 | 100.0 | 100.0 |
| **41-50** | 7 | 1 | 1 | 6 | 87.5 (64.6-100.0) | 85.7 (59.8-100.0) |
| **51-60** | 29 | 0 | 0 | 11 | 100.0 | 100.0 |
| **61-70** | 49 | 0 | 2 | 16 | 100.0 | 88.9 (74.4-100.0) |
| **>70** | 29 | 0 | 0 | 1 | 100.0 | 100.0 |

**Table S14. ROC comparison of the diagnostic performance of the QCIGISH diagnostic grading model and cytology and small biopsy pathology on the model validation set**

| **Diagnostic Performance Comparative Analysis** | | **Computed *p*** |
| --- | --- | --- |
| **Method** | **ROC AUC (95% CI)** |  |
| QCIGISH diagnostic  grading model | 0.98583 (0.96900–1.0000) | **0.03314*** |
| Cytology and small biopsy pathology  (best-case condition) | 0.94411 (0.90790-0.98030) |  |
| QCIGISH diagnostic  grading model | 0.98583 (0.96900–1.0000) | **0.00017*** |
| Cytology and small biopsy pathology  (worst-case condition) | 0.91880 (0.88520-0.95240) |  |

* Computed *p* significant at alpha = 0.05

**Table S15. Comparison of diagnostic sensitivity of QCIGISH and cytology and small biopsy pathology in lung cancer stages.**

| **Lung Cancer Clinical Stage** | **QCIGISH** | | | **Cytology and small biopsy pathology** | | | | **BCC^A^**  ***p*** | **WCC^B^**  ***p*** |
| --- | --- | --- | --- | --- | --- | --- | --- | --- | --- |
|  | **Positive** | **Negative** | **Sensitivity**  **% (95% CI)** | **Positive** | **Indeterminate^C^** | **Negative** | **Sensitivity**  **% (95% CI)** |  |  |
| Stage |  |  |  |  |  |  |  |  |  |
| NSCLC |  |  |  |  |  |  |  |  |  |
| CIS | 1 | 0 | 100.0 | 0 | 0 | 1 | 0.0 (0.0-0.0) | 0.041 | 0.004 |
| Ia | 23 | 1 | 95.8 (87.8-100.0) | 17 | 4 | 3 | 70.8 (52.6-89.0) |  |  |
| Ib | 10 | 0 | 100.0 | 7 | 0 | 3 | 70.0 (41.6-98.4) |  |  |
| II | 15 | 0 | 100.0 | 14 | 0 | 1 | 93.3 (80.7-100.0) | 1.000 | 1.000 |
| III | 17 | 0 | 100.0 | 13 | 4 | 0 | 76.5 (56.3-96.6) | ^D^ | 0.134 |
| IV | 27 | 0 | 100.0 | 24 | 1 | 2 | 88.9 (77.0-100.0) | 0.480 | 0.248 |
| SCLC |  |  |  |  |  |  |  |  |  |
| Limited stage | 11 | 0 | 100.0 | 10 | 0 | 1 | 90.9 (73.9-100.0) | 1.000 | 1.000 |
| Extensive stage | 12 | 0 | 100.0 | 12 | 0 | 0 | 100.0 | ^D^ | ^D^ |
| **All cancers** | 116 | 1 | 99.1 (97.5-100.0) | 97 | 9 | 11 | 82.9 (76.1-89.7) |  |  |

^A^, BCC refers to the best-case condition where the indeterminate cytology and small biopsy cases were classified as positive during diagnostic accuracy comparison with QCIGISH. ^B^, WCC refers to the worst-case condition where the indeterminate cytology and small biopsy cases were classified as negative during diagnostic accuracy comparison with QCIGISH. ^C^, considered as incorrect classification during diagnostic sensitivity computation. ^D^, complete agreement between the results of both diagnostic tests.

**Table S16. Comparison of diagnostic sensitivity of QCIGISH and cytology and small biopsy pathology in lung nodules or masses.**

|  | **QCIGISH** | |  | | **Small tissue pathology** | | |  | **BCC^A^**  ***p*** | | | **WCC^B^**  ***p*** |
| --- | --- | --- | --- | --- | --- | --- | --- | --- | --- | --- | --- | --- |
|  | **Pos.** | **Neg.** | | **Sensitivity,**  **% (95% CI)** | **Malignant** | **Indeterminate^C^** | **Benign** | **Sensitivity,**  **% (95% CI)** |  |  |  |  |
| **Final Diagnosis** | **Malignant** | | |  | **Malignant** | | |  |  |  |  |  |
| **Nodule/Mass size** |  |  | |  |  |  |  |  |  |  |  |  |
| Nodules |  |  | |  |  |  |  |  |  | | |  |
| <2.0 cm | 21 | 0 | | 100.0 | 14 | 4 | 3 | 66.7 (46.5-86.8) |  | 0.248 | | 0.023 |
| >2.0-3.0 cm | 32 | 0 | | 100.0 | 28 | 2 | 2 | 87.5 (76.0-99.0) |  | 0.480 | | 0.134 |
| Masses |  |  | |  |  |  |  |  |  |  | |  |
| >3.0-5.0 cm | 42 | 0 | | 100.0 | 35 | 3 | 4 | 83.3 (72.1-94.6) |  | 0.134 | | 0.023 |
| >5.0 cm | 18 | 0 | | 100.0 | 17 | 0 | 1 | 94.4 (83.9-100.0) |  | 1.000 | | 1.000 |

^A^, BCC refers to the best-case condition where the indeterminate cytology and small biopsy cases were classified as positive during diagnostic accuracy comparison with QCIGISH. ^B^, WCC refers to the worst-case condition where the indeterminate cytology and small biopsy cases were classified as negative during diagnostic accuracy comparison with QCIGISH. ^C^, considered as incorrect classification during diagnostic sensitivity computation. Pos., positive. Neg., negative. N.E.D., no enough data.

**Table S17. Comparison of diagnostic specificity of QCIGISH and cytology and small biopsy pathology in lung nodules or masses.**

|  | **QCIGISH** | |  | | **Small tissue pathology** | | |  | **BCC^A^**  ***p*** | | | **WCC^B^**  ***p*** |
| --- | --- | --- | --- | --- | --- | --- | --- | --- | --- | --- | --- | --- |
|  | **Pos.** | **Neg.** | | **Specificity,**  **% (95% CI)** | **Malignant** | **Indeterminate^C^** | **Benign** | **Specificity,**  **% (95% CI)** |  |  |  |  |
| **Final Diagnosis** | **Benign** | | |  | **Benign** | | |  |  |  |  |  |
| **Nodule/Mass size** |  |  | |  |  |  |  |  |  |  |  |  |
| Nodules |  |  | |  |  |  |  |  |  | | |  |
| <2.0 cm | 1 | 11 | | 91.7 (76.0-100.0) | 0 | 0 | 12 | 100.0 |  | 1.000 | | 1.000 |
| >2.0-3.0 cm | 0 | 3 | | 100.0 | 0 | 0 | 3 | 100.0 |  | ^D^ | | ^D^ |
| Masses |  |  | |  |  |  |  |  |  |  | |  |
| >3.0-5.0 cm | 0 | 4 | | 100.0 | 0 | 0 | 4 | 100.0 |  | ^D^ | | ^D^ |
| >5.0 cm | 0 | 0 | | N.E.D. | 0 | 0 | 0 | N.E.D. |  | ^D^ | | ^D^ |

^A^, BCC refers to the best-case condition where the indeterminate cytology and small biopsy cases were classified as positive during diagnostic accuracy comparison with QCIGISH. ^B^, WCC refers to the worst-case condition where the indeterminate cytology and small biopsy cases were classified as negative during diagnostic accuracy comparison with QCIGISH. ^C^, considered as incorrect classification during diagnostic specificity computation. ^D^, complete agreement between the results of both diagnostic tests. Pos., positive. Neg., negative. N.E.D., no enough data.
